# Circulated echovirus 18 strains in Guangdong Province and worldwide: A novel perspective on genetic diversity and recombination patterns

**DOI:** 10.1080/21505594.2025.2534519

**Published:** 2025-07-15

**Authors:** Zhiyu Li, Xiaohan Yang, Ru Bai, Ping Li, Yanting Qin, Mingyong Luo

**Affiliations:** a Guangzhou Medical University, Guangzhou, China; b Department of Clinical Laboratory, Guangdong Women and Children Hospital, Guangzhou, China; c Women and Children’s Hospital, Southern University of Science and Technology, Guangzhou, China; d Medical Genetic Center, Guangdong Women and Children Hospital, Guangzhou, China

**Keywords:** Echovirus 18, seasonal characteristic, genetic diversity, evolutionary dynamic, recombination patterns

## Abstract

Echovirus 18 (E18) has re-emerged as a global public health concern in recent years because of its association with severe neonatal systemic diseases that pose a risk of high mortality. The lack of effective intervention strategies for E18 infections is largely attributed to limited knowledge regarding molecular epidemiology and recombination patterns. In this study, we obtained seven full-length E18 sequences from infants in Guangdong Province and combined them with representative sequences from GenBank. Using this expanded dataset, we analysed the molecular epidemiological features, genetic characteristics, and recombination patterns of E18. Global statistics reveal a distinct double-peak pattern in the frequency of E18 infections throughout the year in the Northern Hemisphere. All strains isolated from clinical specimens were classified as genotype C2, which has emerged as the predominant circulating strain in Guangdong Province and globally. Several potential recombination events with E30 were identified among these seven strains, particularly in the P2 and P3 non-structural regions. This study offers new insights into the global dissemination, genetic diversity, and phylodynamics of E18, potentially providing valuable information for designing antiviral vaccines and the implementation of sustainable surveillance strategies to enhance virus prevention and control during public health crises.

## Introduction

Human enterovirus (EV) is a crucial pathogen that can cause various illnesses in neonates and young children, ranging from mild to severe conditions, and sometimes life-threatening diseases [1–4]. There are 31 distinct types of enterovirus B (EV-B) (E1–7, 9, 11–21, 24–27, and 29–33) (https://ictv.global/). The increasing frequency of mother-to-child transmission of echoviruses poses significant challenges to prevention and control efforts, despite the primary transmission routes being faecal-oral or respiratory [4]. Recent studies have highlighted that E18 is a re-emerging pathogen responsible for various systemic disorders in neonates, including severe pneumonia, neonatal sepsis, hepatic necrosis with coagulopathy, meningitis, and hand-foot-mouth disease, leading to high mortality rates [5–8]. Therefore, continuous monitoring and rapid diagnosis are important to reduce the risk of post-neonatal mortality and effectively manage and control epidemics.

E18 is a small non-enveloped icosahedral virus belonging to the *Picornaviridae* family and is a positive-sense RNA virus. E18 is grouped into four genotypes (A, B, C1, and C2) based on characteristics of the VP1 gene region [9–11]. The prototypical genome of E18 is approximately 7.4–7.5 kb in length, featuring an open reading frame (ORF) that is flanked by a long 5’ untranslated region (UTR) of approximately 750 nucleotides and a short 3' UTR of approximately 100 nucleotides [12]. The ORF is translated into a large polyprotein that undergoes viral protease cleavage into three protein products: P1, P2, and P3. P1 serves as the precursor of four capsid proteins (VP1–VP4), with VP1 containing multiple immunodominant neutralization epitopes crucial for identifying EV genotypes [13]. P2 and P3 are precursors to non-structural proteins (2A–2C, and 3A–3D), regulating viral RNA replication, viral particle assembly, and host immune response evasion [13].

Continuous monitoring and prompt diagnosis are essential to reduce the risk of post-neonatal mortality and effectively manage and control epidemics. Mutations and recombination events in EVs are key drivers of the emergence of new genotypes, variations in virulence, and their impact on disease progression and outcomes [14]. Previous studies have identified homologous recombination signals and distinct recombination patterns across various EV infections, including E6 in Spain [15], E9 [16], and E30 in China [17]. Therefore, investigating these recombination patterns and mutations in EVs is crucial for understanding both regional and global epidemiological shifts as well as the evolutionary dynamics of these viruses.

A multicenter study on EV infections identified EV-B as the most prevalent aetiological agent in neonates, with E18 ranking among the top three [4]. This finding indicates that E18 has re-emerged as a significant factor in paediatric diseases following a period of low prevalence; however, a significant gap remains in the literature concerning the systematic analysis of the full-length genome variety and recombination forms of E18 on a global scale. This study aimed to bridge this gap by comprehensively exploring the genetic diversity and recombination patterns of E18 strains, utilizing seven newly acquired clinical specimens from Guangdong Province, China, alongside global sequences obtained from the GenBank public database (http://www.ncbi.nlm.nih.gov/genbank/).

## Materials and methods

### Stool sample collection

Stool samples were collected from infants in Guangdong Province between June 2019 and June 2022. These infants were suspected of having sepsis-like conditions, viral meningitis, or other infection-related diseases. The samples utilized were residual specimens from routine clinical viral testing, and all patient information in the research database was fully de-identified to ensure anonymity and confidentiality. Consequently, the requirement for informed consent from patients was waived by the Medical Ethics Committee of Guangdong Women and Children Hospital (Approval No. 202101213). All procedures were conducted in accordance with the guidelines and regulations established by the research ethics committee and adhered to the Declaration of Helsinki.

### Detection and determination of EV

Viral RNA was extracted from all specimens using an automated system (Smart32, DaAn Gene, Guangzhou, China) and the MagPure Universal RNA Precast Kit (Magen Biotechnology, Guangzhou, China). EVs were detected using a commercial pan-EV real-time PCR assay (DaAn Gene, China) at the Medical Genetic Center of Guangdong Women and Children Hospital. All experiments were performed according to the manufacturer’s instructions. Positive specimens were selected for amplification of the VP1 region using pairs of primers 222/224 and AN88/AN89, using a previously described semi-nested PCR method [18]. Finally, the PCR products were sent to Sango Biotech Co., Ltd. in Guangzhou, China, for sequencing. The resulting sequences were subsequently analysed using the NCBI BLAST online bioinformatics tool (https://blast.ncbi.nlm.nih.gov/Blast.cgi).

### Whole genome sequencing of E18

To acquire the complete genomic information of E18, three pairs of specific primers were designed to cover the entire E18 genome using the primer-walking approach (Supplementary Table S1). The PCR products were purified using a Purification Kit (Qiagen, Germany) and sent to Sango Biotech (Guangzhou, China) for further sequencing. Finally, the seven complete genome sequences determined in this study were assembled and deposited in GenBank (accession numbers: PP891437–PP891443).

### Construction of datasets for Chinese and global E18

A total of 703 nucleotide sequences, each associated with a specific country or region and precise date, were retrieved from GenBank (before 1 August 2023) to analyze the molecular epidemiological and evolutionary patterns of E18. Ultimately, 588 sequences were retained in the initial dataset for analysis after removing problematic or low-quality sequences, including two prototype strains. Subsequently, 342 representative strains isolated between 1997 and 2022 were selected to investigate potential seasonal patterns. To investigate the distribution of isolated sources, a novel dataset, designated as the 256 dataset, was created using various sample types (e.g., stool, rectal swab, and cerebrospinal fluid [CSF]) derived from the initial dataset. Sequences with complete VP1 regions were compiled into a new dataset, referred to as the 290 dataset, for evolutionary analysis. A dataset comprising seven complete genome sequences from Guangdong, China, along with 63 full-length sequences sourced from the GenBank database, collectively referred to as the 70 dataset, was constructed for phylogenetic analysis. To facilitate recombination analysis, Neighbor-Joining (NJ) phylogenetic trees were constructed separately for the P1, P2, and P3 regions of the E18 genomes, as well as for closely related E30 strains. The detailed information on all datasets is provided in Supplementary File 1: Tables S2–S9.

### Phylogenetic analysis and estimation of evolutionary rate

E18 sequences were aligned using MAFFT software (v.7.4) [19]. The best nucleotide substitution model, as determined by ModelTest-NG, was GTR + G4, which yielded the highest BIC scores among all runs [20]. Phylogeny analysis and estimation of evolutionary rates (substitutions/site/year) were performed using the Markov chain Monte Carlo (MCMC) approach in the BEAST software (v1.10.4) based on the complete VP1 gene of E18 globally [21]. An uncorrelated relaxed clock with exponential growth was applied, running for 100 million generations with a sampling frequency of 10,000 states. The convergence and effective sample size of the parameters were assessed using TRACER (v.1.7.1). A maximum clade credibility tree (MCC) was generated using TreeAnnotator (v.1.10.4) after discarding the initial 10 % as burn-in, and the output result was visualized using FigTree (v.1.4.4). Furthermore, phylogenetic analysis was conducted using MEGA software (v.11.0) with the NJ method and 1000 re-sampling strategy, focusing on coding genes (P1, P2, and P3) and the complete genomic sequences of E18 [22].

### Detection of recombination lineage

The complete genomic sequences of E18 were examined for potential recombinants using GenBank. Specifically, seven P2 and P3 non-structural protein region strains were analysed using BLAST to compare their identities with sequences from the public GenBank database. The consensus criterion for grouping EVs is a minimum divergence of 25 % in the complete genome of the VP1 region [9]. Accordingly, sequences with similarities of VP1 ≥ 85 % were identified as potential parents of the seven strains from Guangdong Province. In comparison, other EV-B sequences with similarities < 75 % were considered potential recombination candidates and were subsequently downloaded from GenBank. Similarity plots were constructed and boot-scanning analyses were performed to identify recombination breakpoints using the Simplot software (v3.5.1), employing a 200-nucleotide sliding window that advanced in 20-nucleotide increments with 500 replications [12,17]. The genetic distance of E18 was calculated using the Kimura two-parameter model via MEGA, with 1000 bootstrap replicates. Evolutionary divergence within the 5’ UTR, P1 (VP1–VP4), P2 (2A–2C), and P3 (3A–3D) among the selected seven strains and 70 representative reference strains from various countries was assessed using MEGA [22]. A heatmap was constructed to visualize the hereditary distances between E18 segments.

## Results

### Overview of E18 strains isolated from clinical samples in Guangdong Province

Seven clinical samples that tested positive for E18 infection were collected from six cities across Guangdong, China, and included in this analysis. Among these, four (4/7, 57.1 %) samples were isolated from male participants, five (5/7, 71.4 %) were from neonates aged 0–28 d, and two (2/7, 28.6 %) were from infants aged 29 d–3 months. Of the four patients with confirmed diagnoses, three presented with pneumonia and one with purulent meningitis. The most common early-onset symptom was fever, often accompanied by complications such as anaemia and jaundice. The detailed information of patients was listed in Supplementary File 1: Table S10.

### District distribution of E18 in China and worldwide

**Figure 1.**
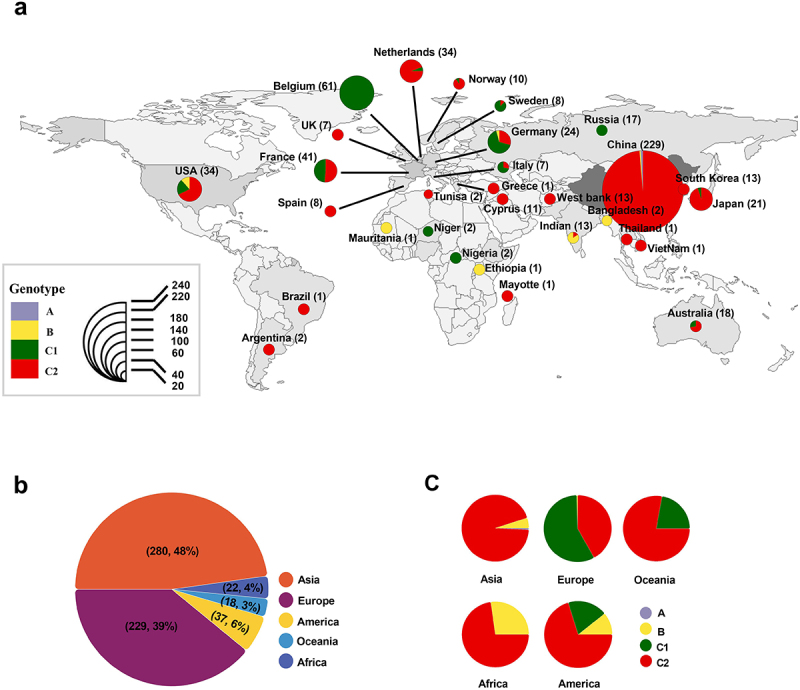
District distribution of E18 in Guangdong Province and worldwide. (a) the district distribution of E18 globally from 1955 to 2022. The pie charts illustrate the frequency of virus genotypes by location, with numbers in brackets indicating the quantity of the virus identified in each location. (b) The percentage distribution of the virus across different continents. (c) The distribution of viral genotype among continents.

Our sequences, combined with those available from GenBank, comprised a dataset of 588 sequences used to investigate the epidemiology of E18. The global frequency and spatial distribution of E18 are shown in Figure 1. E18 strains were isolated from 1955 (prototype strain Metcalf) to 2022 across 29 countries and regions, indicating widespread spatiotemporal and regional distribution over an extended period (Figure 1(a)). These regions encompassed Asian, European, North American, South American, Australian, and African countries (Figures 1(b,c) and Table 1). Among the identified E18-associated infections, 47.8 % (280/586) originated in Asia, and 39.1 % (229/586) were reported in Europe (Figure 1(b)). During the global dissemination of E18, the Asia-Pacific region emerged as a significant phylogenetic trunk location, with China accounting for the largest proportion of cases (81.8%, 229/280). E18 was initially identified in 2000 in Yunnan Province, China, and subsequently spread rapidly across several southern regions, including Jiangsu, Zhejiang, Guangdong, and Hunan. In northern regions of China, provinces such as Hebei and Shandong reported higher infection rates, primarily due to viral encephalitis pandemics that occurred in 2015 and 2019 (Supplementary File 2: Supplemental Figure S1).

**Table 1.** The distribution of the E18 genotype across different countries and continents.

			Genotype of E18			
Continent	Country	Total	A	B	C1	C2
Asia		280	2	14	0	264
	China	229	2	1	0	216
	Japan	21	0	0	0	21
	India	13	0	11	0	2
	South Korea	13	0	0	0	13
	Thailand	1	0	0	0	1
	Bangladesh	2	0	2	0	0
	Vietnam	1	0	0	0	1
Europe		229	0	1	132	96
	Belgium	61	0	0	61	0
	France	41	0	0	20	21
	Netherlands	34	0	0	2	32
	Germany	24	0	1	16	7
	Russia	17	0	0	17	0
	Cyprus	11	0	0	0	11
	Sweden	8	0	0	7	1
	Norway	10	0	0	1	9
	Italy	7	0	0	5	2
	Spain	8	0	0	3	5
	UK	7	0	0	0	7
	Greece	1	0	0	0	1
Africa		22	0	6	0	16
	West Bank	13	0	0	0	13
	Ethiopia	1	0	1	0	0
	Niger	2	0	2	0	0
	Nigeria	2	0	2	0	0
	Mauritania	1	0	1	0	0
	Tunisia	2	0	0	0	2
	Mayotte	1	0	0	0	1
America		37	0	4	7	26
	USA	34	0	4	7	23
	Argentina	2	0	0	0	2
	Brazil	1	0	0	0	1
Oceania		18	0	0	4	14
	Australia	18	0	0	4	14

USA: the United States of America; UK: the United Kingdom.

### Temporal distribution and seasonality of E18

**Figure 2.**
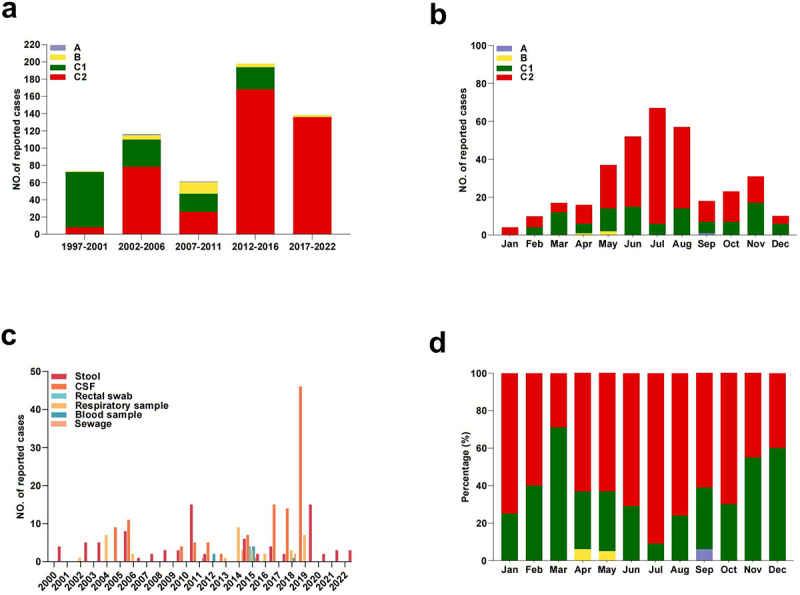
The temporal distribution and seasonality characteristics of global E18. (a) the yearly distribution of E18. (b) The monthly distribution of E18. (c) The distribution of isolated sources of E18. (d) The monthly percentage proportion of E18.

To capture the temporal distribution and seasonal characteristics of E18 virus lineages, a global dataset of E18 genetic sequences was assembled, dating back to 1997 (see Materials and Methods). The data revealed significant fluctuations in circulation patterns over various periods, with a notable surge observed before 2016, followed by a slight decreasing trend (Figure 2(a)). Before 2001, the majority of E18 viral strains were classified as belonging to the C1 genotype. However, after 2001, the C2 genotype became dominant and played a crucial role in the global dissemination of echoviruses. From 2002 to 2016, four genotypes co-circulated, with genotype B emerging in 2002 and exhibiting a slight upward trend thereafter. The monthly frequency of E18 infection cases, along with their proportional distributions, revealed a distinct seasonal pattern in the global epidemic each year (Figure 2(b)). In the northern hemisphere, the peak activity period was predominantly observed during warmer months, specifically from April to August, with July recording the highest number of infections at 19.6 % (67/342). A minor peak was observed from September to November. Few cases were reported during the winter, suggesting a facultative bimodal seasonal pattern of E18 infection.

Current data indicate that stool, CSF, and respiratory specimens account for > 95 % of these sequences, underscoring the importance of collecting optimal samples to detect viral shedding (Figure 2(c)). The proportional distribution of genotype is shown in Figure 2(d). Genotype A was rarely detected between April and November, whereas genotype B was consistently detected at low levels throughout the year. Genotype C1 was detected throughout the year, whereas genotype C2 was predominantly observed in spring and summer. Furthermore, an alternating prevalence pattern of C1 and C2 was noted, suggesting the potential influence of seasonal and climatic factors on E18 infection.

### Phylogeny analysis and evolutionary dynamics of global E18

**Figure 3.**
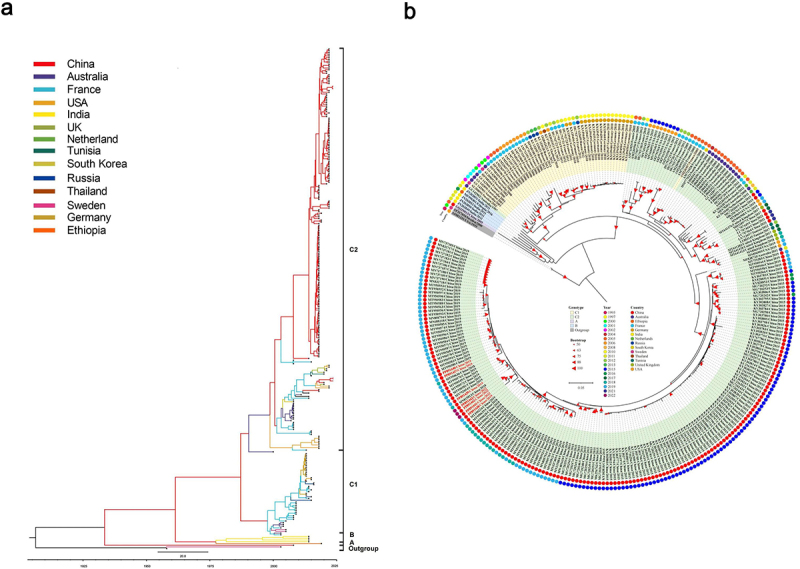
Dynamics of genetic diversity of echovirus 18 lineages. (a) Maximum clade credibility (MCC) tree based on the VP1 gene of E18. The branch colours represent the sampling location of each virus sequence. (b) a neighbour-joining phylogenetic tree for VP1 was constructed using MEGA software version 11.0, with bootstrap values calculated according to the default parameters. Sequences originating from Guangdong Province are highlighted in red, while circles of varying colours represent different locations and years.

**Figure 4.**
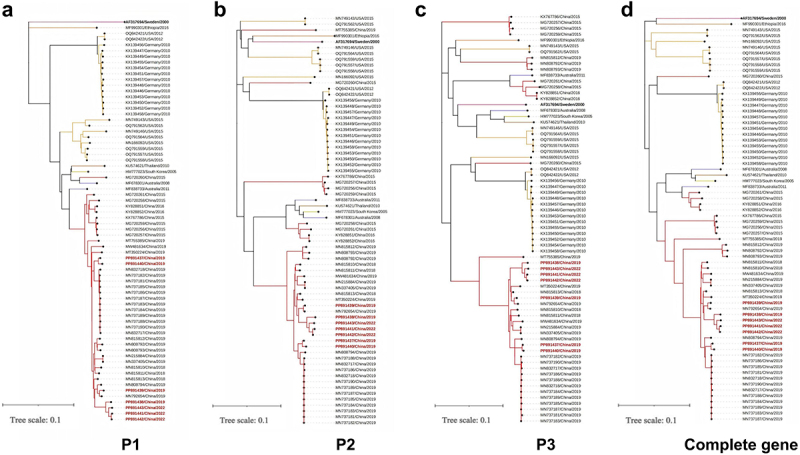
Neighbor-joining phylogeny trees of P1 (a), P2 (b), P3 (c), and the complete genome (d) of E18. Each strain is colour-coded according to its country of isolation. The prototype strain is highlighted in bold black, while the sequences newly sequenced in this study are emphasized in bold red. The scale bars indicate the nucleotide substitutions per site.

The global history of E18 was reconstructed through phylogeny analysis of the complete VP1 gene sampled from Guangdong and worldwide to investigate the genetic diversity, transmission, and evolutionary origin of the viral strains (Figure 3). Dendrograms were constructed using the Bayesian method in the BEAST program (Figure 3(a)), which were aligned with the topological structure of the NJ tree presented in Figure 3(b). Consistent with the previous classifications, the topology of the phylogenetic tree revealed four distinct genotypes, excluding the prototype Metcalf strain (AF081331). This phylogenetic analysis elucidated the evolutionary dynamics of VP1 sequences on a global scale, highlighting the shifts in circulating strains across 14 countries. The countries included in this study were mainland China (*n* = 186), India (*n* = 4), South Korea (*n* = 1), Thailand (*n* = 1), France (*n* = 33), Germany (*n* = 16), Sweden (*n* = 3), the UK (*n* = 4), the Netherlands (*n* = 2), Tunisia (*n* = 1), Ethiopia (*n* = 1), Russia (*n* = 4), Australia (*n* = 18), and the USA (*n* = 15). The genetic distances between the genotypes ranged from 13.5 % to 25.0 %, indicating distinct groupings. The prototype strain, first isolated in the USA in 1955, formed an outgroup with a strain isolated in Sweden in 2000. Genotype A was represented by a single strain isolated in China in 2005, whereas genotype B included strains isolated in India in 2011 and Ethiopia in 2016. Most C1 strains were isolated in Europe, whereas C2 strains were identified in eight countries, with a notable concentration in China, suggesting global dissemination. Most notably, the newly sequenced samples derived from Guangdong clustered within the C2 group, exhibiting a high degree of similarity in the complete VP1 region, with nucleotide identities ranging from 95.2 % to 99.6 %, and amino acid identities ranging from 82.0 % to 100 %. The maximum clade credibility (MCC) tree indicated that the average evolutionary rate of the VP1 gene was 3.46 × 10^−3^ (95 % HPD: 1.50 × 10^−4^–1.51 × 10^−4^) substitutions per site per year. Figure 4 showed the NJ phylogenetic trees of E18 strains originating from China and other countries. These trees were constructed separately based on the P1 (Figure 4(a)), P2 (Figure 4(b)), and P3 (Figure 4(c)) regions, in addition to the complete genome (Figure 4(d)).

### Detection of genetic recombination breakpoints in the E18 genomes

**Figure 5.**
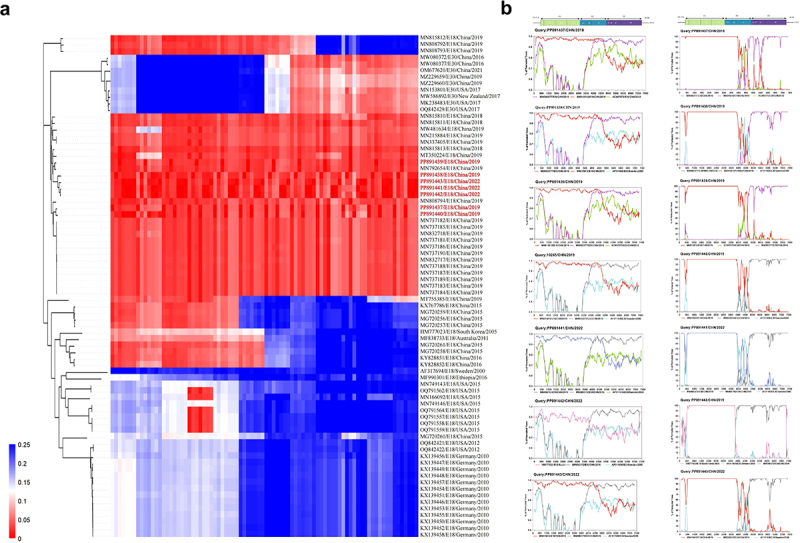
Analysis of recombination patterns. (a) The phylogeny tree and heatmap were constructed based on the sequences obtained in this study, along with representative sequences available from the GenBank database. Sequences isolated from Guangdong, China, are highlighted in red. Positive correlations are indicated by blue squares, while negative correlations are represented by red squares within the heatmap regions. (b) Similarity and bootscanning analyses were conducted using a sliding window of 200 nucleotides, moving in steps of 20 nucleotides, with 500 replicates. RNA recombination breakpoints were frequently observed in the P1 and P2 regions.

Phylogenetic analysis, similarity plotting, and bootscanning analyses were conducted on the protein precursors of E18 to explore the relationship between recombination events and viral evolution (Figure 5). The results revealed significant topological differences in the phylogenetic trees of the E18 genomes compared to those of closely related E30 strains. The phylogenetic tree constructed based on the P1 sequences indicated that the seven strains analysed in this study exhibited greater evolutionary distances from the E30 strain (Supplementary File 2: Supplemental Figure S2a). Conversely, the phylogenetic analysis of the P2 and P3 regions revealed that all E18 strains maintained a closer phylogenetic relationship with the E30 strain compared to the P1 region, suggesting the occurrence of potential recombination events in these regions (Supplementary File 2: Supplemental Figure S2b,c). Moreover, the newly sequenced E18 strains in this study exhibited a high degree of nucleotide similarity in the P2 and P3 regions, ranging from 86.9% to 90.1% and from 91% to 95.5%, respectively. However, they displayed lower similarity in the P1 region (55.1%-56.0%).

To accurately examine the presence of recombination events and the circulation trends of recombinant strains in each epidemic in China and worldwide, a heatmap was constructed to assess the evolutionary diversity of global E18 strains (Figure 5(a)). Numerous recombinant E18 strains have evolved continuously and have persisted for many years, demonstrating a broad global distribution. Prior to 2016, the predominant recombinant strain was genotype C1. Subsequently, genotype C2 gradually became the dominant circulating recombinant strain, replacing C1 during later outbreaks in mainland China. These changes may be influenced by various factors, including environmental conditions, geographic movements, and demographic shifts. Notably, all seven E18 strains examined were classified as C2 recombinant viruses. The P2 and P3 regions of the viral genome exhibited low similarity to the prototype Metcalf strain (AF317694). However, the 2C and 3A–3D regions of E18 showed minimal evolutionary differences compared to those of the E30 strains (MW080377, MW080372, and OM677620). The findings from the bootscanning analysis, in conjunction with the previous Simplot analysis, provided strong evidence for multiple recombination events in both the E18 and E30 strains (Figure 5(b)).

## Discussion

E18 has been identified as a re-emerging causative agent of both sporadic cases and epidemic outbreaks in neonates [4,7]. Numerous studies have highlighted its critical role in severe neonatal disorders across Europe [23,24], Asia [11], America [25], and Oceania [26]. Therefore, ongoing surveillance and evaluation of the genetic diversity of circulating E18 strains are essential for mitigating large-scale pandemics and reducing mortality rates. This study presents a systematic analysis of E18 strains isolated between 1955 and 2022, alongside seven complete genome sequences obtained from Guangdong Province, offering novel insights into the molecular epidemiological characteristics, genetic diversity, and recombination patterns of E18.

E18 has rapidly spread globally and has become a critical aetiological factor in severe disorders. To better represent the diversity, 586 E18 strains from across the world were analysed for epidemiological distribution. Our data revealed a global upsurge in E18 infections, with a particularly high prevalence observed in the coastal regions of Europe and landlocked countries in west-central Asia. Belgium, France, and the Netherlands emerged as the top three nations in Europe with the most E18 cases identified. This is consistent with previous surveys on circulating EVs in European countries, such as the study conducted by Bubba et al, which indicated significant echovirus activity in 2015–2017 [27]. In Asia, China has seen a considerable spread and increase in E18-related infections, in contrast to several other countries, such as South Korea, Japan, and India, where infections are more sporadic. Furthermore, several studies have documented several outbreaks of E18-induced severe illnesses, including meningitis in Alaska [25], Germany [28], New Zealand [26], Japan [29], and China [30], thereby highlighting the dispersed patterns of global E18 to substantial transmission. This resurgence of E18 infection worldwide may be influenced by the heterogeneity of the population as well as the multiplicity of the environment.

Our study focused on yearly variations in the number of E18 cases and highlighted the seasonal patterns of circulating strains over different periods. From 1997 to 2022, E18 infection cases exhibited a double upsurge pattern, with notable peaks occurring before and after 2011 and 2017, respectively. This significant variation may be attributed to several factors, including the large-scale outbreak of CVA6 in Finland in 2008 [31]; the emergence of E9, E30, CVB5, and EV-D68 in the USA from 2017 to 2022 [32]; and the development of multiple EV-A71 vaccines to aimed at combating EV-A71-associated diseases, which led to shifts in the predominant EV pathogen [33]. Another factor may be the outbreak of severe acute respiratory syndrome coronavirus in 2019, along with variation in human behaviour driven by the implementation of nonpharmaceutical interventions (NPIs) in response to the COVID-19 pandemic in 2020, which diverted public health attention from the ongoing surveillance of enteroviruses [34,35].

The prevalence of enterovirus is influenced by various factors, including natural elements such as season, latitude, and climate [36]. In the Northern Hemisphere, areas affected by E18 infections showed a distinct bimodal pattern in seasonal occurrence, which is consistent with other findings on non-polio enteroviruses, such as coxsackievirus A6 (CVA6) and echovirus E11 [37,38]. Echovirus 18 demonstrates its highest abundance from April to August, with a clear upward trend in early summer and a declining trend noted in early winter, consistent with previous studies on enterovirus 71 (EV-A71) infections [39]. The reasons for this variation in circulation patterns remain unclear, underscoring the need for further studies to elucidate the precise mechanisms underlying this phenomenon.

Understanding the phylogenetics of E18 is essential for a comprehensive grasp of its evolutionary history. The evolving circulation of E18 strains highlights how the virus has developed distinct regional characteristics through long-term evolution and adaptation. Notably, genotype C2 transmission re-emerged in 2015 following the outbreak of E18 infection in China, while genotype A stopped circulating after 2005. The reduction in relative genetic diversity was slightly less pronounced for genotype A viruses, as multiple genotypes continued to co-circulate from 2006 to 2022. Our findings found that the evolution rate of E18 continuously changed over time, suggesting the frequent occurrence of mutations and recombination phenomena. This phenomenon is consistent with previous studies on EVs, including Coxsackievirus A16 (2.49 × 10^−3^ substitutions/site/year) [40], EV71 (4.6 × 10^−3^ substitutions/site/year) [41], E5 (7.74 × 10^−3^ substitutions/site/year) [42], and CVB3 (4.82 × 10^−3^ substitutions/site/year) [43]. Thus, surveillance of the genetic diversity of E18 is essential for improving our understanding of epidemic trends and the geographic dynamic spread of these viruses. This comprehension is crucial for developing more effective therapeutic interventions and antiviral drugs to prevent larger-scale virus transmission.

Recombination is the primary driving force of viral adaptation, genetic variation, host transmission, and phylodynamic inference [36]. Research on EV-B infections in neonates in China revealed evidence of recombination between the E6 and E11 types [33]. The co-circulation of CVA16 and EV71 influences EV evolution [8]. Enteroviruses exhibit relatively high mutation and evolution rates, primarily because of the absence of a proofreading mechanism in their RNA-dependent RNA polymerase [44]. Evidence indicates that recombination breakpoints are typically located in the non-structural regions of EV, particularly within the P2 and P3 regions [12]. Additionally, seven complete genomes were analysed, and a significant recombination event was identified involving large segments in the P2 and P3 regions, particularly those associated with the E30 virus strains from China. This variation in recombination may be a key factor contributing to the increased infectivity or virulence of the virus. Consequently, the precise detection and identification of recombination events are essential for understanding emerging pathogens, which is vital for mitigating the associated risk of large-scale epidemic transmission.

Despite this study providing valuable insights, it also had several limitations. Previous research has shown that E18 can lead to severe and fatal illnesses in neonates, with significant variability in virulence levels among different strains within the same genotype. The age of the infected individual notably influences the severity of symptoms; generally, older children exhibit milder symptoms and a lower risk of complications compared to newborns. However, this study was limited to specimens from Guangdong Province, where the epidemiological characteristics may differ from those observed in foreign countries. Challenges in obtaining detailed clinical data have hindered robust statistical analyses to evaluate the relationship between genotype and clinical outcomes. Furthermore, the relatively small sample size used for phylogeny analysis may have introduced bias regarding the spatiotemporal distribution and current epidemiological trends. It is also important to acknowledge that, before 2001, the limited availability of sequencing technologies and genotyping tools likely resulted in many undetected strains or only partially characterized. Consequently, genotype C1 May have appeared more geographically restricted, potentially introducing bias in early surveillance data. Additionally, the limited availability of complete genome sequences in public databases, combined with a predominant focus on analysing the evolutionary dynamics of the VP1 region, restricted a comprehensive exploration of E18. Lastly, deficiencies in E18 surveillance data impeded the investigation of recombination events, which are crucial for further understanding pathogenicity and the circulating patterns of recombination from a public health perspective.

## Conclusion

This study substantiates and extends previous research on the increase in EV-B infections among the infant population from 2019 to 2022, highlighting the emergence of E18-associated fatal systemic infectious diseases in multiple countries and prompting enhanced vigilance. The genetic diversity of global E18 sequences was analysed based on ORF regions, providing novel insights into current epidemiological trends and evolutionary dynamics. Furthermore, our findings identified multiple recombination events with E30 among C2 strains from Guangdong Province that have shown global dissemination, underscoring the necessity for ongoing monitoring of evolutionary diversity changes and the identification of emerging viral strains that may increase virulence and transmissibility. This study serves as a valuable guide for the development of multivalent vaccines against E18 and the implementation of sustainable and effective prevention and control strategies.

## Supplementary Material

Supplemental Material

## Data Availability

Raw and processed data have been deposited in the GenBank database at NCBI under the accession numbers PP891437 to PP891443 (https://www.ncbi.nlm.nih.gov/genbank/). Additionally, the data and supplementary materials supporting the findings of this study are publicly available on Figshare at https://doi.org/10.6084/m9.figshare.27908790.v10.
