# Supplementary material for: Circulated echovirus 18 strains in Guangdong Province and worldwide: A novel perspective on genetic diversity and recombination patterns
Source: Virulence. 2025 Jul 15;16(1):2534519. doi: 10.1080/21505594.2025.2534519 (PMC12296116; doi:10.1080/21505594.2025.2534519)
Supplement: Supplemental Material [file KVIR_A_2534519_SM5328.zip › Supplementary File_1_Table_S1.docx]

**Supplementary Table S1.** Primers used in the present study for complete genome amplification of E18

| Name | Product length | Sequence (5'-3') | Orientation |
| --- | --- | --- | --- |
| E18-F1 | 2574 | CAGCCTGTGGGTTGTTCC | Forward |
| E18-R1 |  | AGTTTGTAGGGTGTCGCTRG | Reverse |
| E18-F2 | 2768 | CTGCATGCAACGACTTCTCG | Forward |
| E18-R2 |  | GACCGCCTCACTATCCACTG | Reverse |
| E18-F3 | 2369 | GACCGCCCGCATATAGAGAG | Forward |
| E18-R3 |  | GCACCGAATGCGGAGAATTT | Reverse |
| ^a^ Numbering according to the full-length genome of echovirus 18 strain BJ2018-S6363 (GenBank accession number: MN815811.1). | | | |
