# Supplementary material for: Circulated echovirus 18 strains in Guangdong Province and worldwide: A novel perspective on genetic diversity and recombination patterns
Source: Virulence. 2025 Jul 15;16(1):2534519. doi: 10.1080/21505594.2025.2534519 (PMC12296116; doi:10.1080/21505594.2025.2534519)
Supplement: Supplemental Material [file KVIR_A_2534519_SM5328.zip › Supplementary File_1_Table_S10.docx]

**Supplementary Table S10 .** The **clinical characteristics and epidemiological data** of seven E18 strains **isolated in Guangdong Province between 2019 and 2022.**

| Cases | Sex | Age | Region | Clinical diagnosis | Isolated source |
| --- | --- | --- | --- | --- | --- |
| Case 1 | female | 28 days | Guangzhou | purulent meningitis | stool |
| Case 2 | male | 21 days | Yunfu | pneumonia | stool |
| Case 3 | male | 3 months | Guangzhou | unknown | stool |
| Case 4 | male | 33 days | Shenzhen | unknown | stool |
| Case 5 | female | 23 days | Huizhou | sepsis-like disease, pneumonia | stool |
| Case 6 | female | 21 days | Huizhou | encephalitis, pneumonia | stool |
| Case 7 | male | 7 days | Yunfu | unknown | stool |
