# Supplementary material for: Circulated echovirus 18 strains in Guangdong Province and worldwide: A novel perspective on genetic diversity and recombination patterns
Source: Virulence. 2025 Jul 15;16(1):2534519. doi: 10.1080/21505594.2025.2534519 (PMC12296116; doi:10.1080/21505594.2025.2534519)
Supplement: Supplemental Material [file KVIR_A_2534519_SM5328.zip › Supplementary File_1_Table_S2.docx]

**Supplementary Table S2.** Information of 588 E18 sequences in Guangdong Province and worldwide.

| Accession | Year | Country | Source | Genotype |
| --- | --- | --- | --- | --- |
| PP891437 | 2019 | China | This study | C2 |
| PP891438 | 2019 | China | This study | C2 |
| PP891439 | 2019 | China | This study | C2 |
| PP891440 | 2019 | China | This study | C2 |
| PP891441 | 2022 | China | This study | C2 |
| PP891442 | 2022 | China | This study | C2 |
| PP891443 | 2022 | China | This study | C2 |
| AB167997 | 2001 | Japan | GenBank | C2 |
| AB167998 | 2001 | Japan | GenBank | C2 |
| AB167999 | 2001 | Japan | GenBank | C2 |
| AB168000 | 2001 | Japan | GenBank | C2 |
| AB188509 | 2004 | Japan | GenBank | C2 |
| AB199309 | 2004 | Japan | GenBank | C2 |
| AB199310 | 2004 | Japan | GenBank | C2 |
| AB199311 | 2004 | Japan | GenBank | C2 |
| AB199312 | 2004 | Japan | GenBank | C2 |
| AB199313 | 2004 | Japan | GenBank | C2 |
| AB199314 | 2004 | Japan | GenBank | C2 |
| AB234348 | 2005 | Japan | GenBank | C2 |
| AB268227 | 2000 | China | GenBank | B |
| AB268228 | 2000 | China | GenBank | C2 |
| AB698775 | 2000 | Japan | GenBank | C2 |
| AB902836 | 2012 | Japan | GenBank | C2 |
| AB920409 | 2013 | Japan | GenBank | C2 |
| AF081602 | 1999 | USA | GenBank | C2 |
| AF081638 | 1999 | USA | GenBank | C1 |
| AF152269 | 1999 | USA | GenBank | C1 |
| AF152282 | 1999 | USA | GenBank | C1 |
| AF152284 | 1999 | USA | GenBank | C1 |
| AF152292 | 1999 | USA | GenBank | C1 |
| AF252159 | 2000 | Spain | GenBank | C1 |
| AF252187 | 2000 | Spain | GenBank | C1 |
| AF252188 | 2000 | Spain | GenBank | C1 |
| AF295436 | 2000 | Sweden | GenBank | C1 |
| AF295468 | 2000 | Sweden | GenBank | C1 |
| AF521383 | 2000 | Belgium | GenBank | C1 |
| AF521384 | 2000 | Belgium | GenBank | C1 |
| AM236918 | 2005 | France | GenBank | C2 |
| AM236953 | 2005 | France | GenBank | C2 |
| AM236956 | 2005 | France | GenBank | C2 |
| AM236957 | 2005 | France | GenBank | C1 |
| AM236970 | 2005 | France | GenBank | C1 |
| AM236972 | 2005 | France | GenBank | C1 |
| AM236978 | 2005 | France | GenBank | C1 |
| AM236984 | 2005 | France | GenBank | C2 |
| AM492410 | 2005 | France | GenBank | C2 |
| AM492411 | 2005 | France | GenBank | C2 |
| AM492412 | 2005 | France | GenBank | C2 |
| AM492413 | 2005 | France | GenBank | C2 |
| AM492414 | 2002 | France | GenBank | C2 |
| AM711021 | 2005 | France | GenBank | C2 |
| AM711024 | 2005 | France | GenBank | C2 |
| AM711028 | 2005 | France | GenBank | C2 |
| AM711068 | 2006 | France | GenBank | C1 |
| AM711070 | 2006 | France | GenBank | C1 |
| AM711073 | 2006 | France | GenBank | C1 |
| AM711101 | 2006 | France | GenBank | C1 |
| AM711103 | 2006 | France | GenBank | C1 |
| AM711105 | 2006 | France | GenBank | C1 |
| AY208090 | 2002 | Sweden | GenBank | C1 |
| AY208091 | 2002 | Sweden | GenBank | C1 |
| AY208092 | 2002 | Sweden | GenBank | C1 |
| AY208093 | 2002 | Sweden | GenBank | C1 |
| AY208094 | 2002 | Sweden | GenBank | C1 |
| AY342659 | 2001 | Belgium | GenBank | C1 |
| AY342660 | 2001 | Belgium | GenBank | C1 |
| AY342663 | 2001 | Belgium | GenBank | C1 |
| AY342673 | 2001 | Belgium | GenBank | C1 |
| AY342675 | 2001 | Belgium | GenBank | C1 |
| AY342676 | 2001 | Belgium | GenBank | C1 |
| AY342677 | 2001 | Belgium | GenBank | C1 |
| AY342678 | 2001 | Belgium | GenBank | C1 |
| AY342679 | 2001 | Belgium | GenBank | C1 |
| AY342688 | 2001 | Belgium | GenBank | C1 |
| AY342689 | 2001 | Belgium | GenBank | C1 |
| AY342690 | 2001 | Belgium | GenBank | C1 |
| AY342747 | 2001 | Belgium | GenBank | C1 |
| AY342748 | 2001 | Belgium | GenBank | C1 |
| AY342749 | 2001 | Belgium | GenBank | C1 |
| AY342750 | 2001 | Belgium | GenBank | C1 |
| AY342751 | 2001 | Belgium | GenBank | C1 |
| AY342752 | 2001 | Belgium | GenBank | C1 |
| AY342753 | 2001 | Belgium | GenBank | C1 |
| AY342754 | 2001 | Belgium | GenBank | C1 |
| AY342755 | 2001 | Belgium | GenBank | C1 |
| AY342756 | 2001 | Belgium | GenBank | C1 |
| AY342757 | 2001 | Belgium | GenBank | C1 |
| AY342758 | 2001 | Belgium | GenBank | C1 |
| AY342759 | 2001 | Belgium | GenBank | C1 |
| AY342760 | 2001 | Belgium | GenBank | C1 |
| AY342761 | 2001 | Belgium | GenBank | C1 |
| AY342762 | 2001 | Belgium | GenBank | C1 |
| AY342763 | 2001 | Belgium | GenBank | C1 |
| AY342764 | 2001 | Belgium | GenBank | C1 |
| AY342765 | 2001 | Belgium | GenBank | C1 |
| AY342766 | 2001 | Belgium | GenBank | C1 |
| AY342767 | 2001 | Belgium | GenBank | C1 |
| AY342768 | 2001 | Belgium | GenBank | C1 |
| AY342769 | 2001 | Belgium | GenBank | C1 |
| AY342770 | 2001 | Belgium | GenBank | C1 |
| AY342771 | 2001 | Belgium | GenBank | C1 |
| AY342772 | 2001 | Belgium | GenBank | C1 |
| AY342773 | 2001 | Belgium | GenBank | C1 |
| AY342774 | 2001 | Belgium | GenBank | C1 |
| AY342775 | 2001 | Belgium | GenBank | C1 |
| AY342776 | 2001 | Belgium | GenBank | C1 |
| AY342777 | 2001 | Belgium | GenBank | C1 |
| AY342778 | 2001 | Belgium | GenBank | C1 |
| AY342780 | 2001 | Belgium | GenBank | C1 |
| AY342782 | 2001 | Belgium | GenBank | C1 |
| AY342783 | 2002 | Belgium | GenBank | C1 |
| AY342790 | 2002 | Belgium | GenBank | C1 |
| AY342797 | 2002 | Belgium | GenBank | C1 |
| AY342799 | 2002 | Belgium | GenBank | C1 |
| AY342800 | 2002 | Belgium | GenBank | C1 |
| AY342805 | 2002 | Belgium | GenBank | C1 |
| AY342806 | 2002 | Belgium | GenBank | C1 |
| AY342810 | 2002 | Belgium | GenBank | C1 |
| AY342815 | 2002 | Belgium | GenBank | C1 |
| AY342830 | 2002 | Belgium | GenBank | C1 |
| AY342831 | 2002 | Belgium | GenBank | C1 |
| AY342832 | 2002 | Belgium | GenBank | C1 |
| AY342833 | 2002 | Belgium | GenBank | C1 |
| AY919455 | 2005 | USA | GenBank | B |
| AY919540 | 2005 | USA | GenBank | B |
| AY919557 | 2005 | USA | GenBank | B |
| AY919563 | 2005 | USA | GenBank | B |
| DQ246765 | 2005 | Argentina | GenBank | C2 |
| DQ246767 | 2005 | Argentina | GenBank | C2 |
| DQ317195 | 2003 | Norway | GenBank | C2 |
| DQ317196 | 2003 | Norway | GenBank | C2 |
| DQ317197 | 2003 | Norway | GenBank | C2 |
| DQ317198 | 2003 | Norway | GenBank | C2 |
| DQ317199 | 2002 | Norway | GenBank | C2 |
| DQ317200 | 2003 | Norway | GenBank | C2 |
| DQ317201 | 2002 | Norway | GenBank | C2 |
| DQ317202 | 2002 | Norway | GenBank | C2 |
| DQ317203 | 2002 | Norway | GenBank | C1 |
| DQ317204 | 2002 | Norway | GenBank | C2 |
| EU077501 | 2007 | Netherlands | GenBank | C2 |
| EU077502 | 2007 | Netherlands | GenBank | C2 |
| EU077505 | 2007 | Netherlands | GenBank | C2 |
| EU077522 | 2007 | Netherlands | GenBank | C2 |
| EU372170 | 2006 | Spain | GenBank | C2 |
| EU372171 | 2006 | Spain | GenBank | C2 |
| EU372172 | 2006 | Spain | GenBank | C2 |
| EU372173 | 2006 | Spain | GenBank | C2 |
| EU372174 | 2006 | Spain | GenBank | C2 |
| EU590818 | 2005 | South Korea | GenBank | C2 |
| EU590819 | 2005 | South Korea | GenBank | C2 |
| EU590820 | 2005 | South Korea | GenBank | C2 |
| EU590821 | 2005 | South Korea | GenBank | C2 |
| EU590822 | 2005 | South Korea | GenBank | C2 |
| EU590823 | 2005 | South Korea | GenBank | C2 |
| EU590824 | 2005 | South Korea | GenBank | C2 |
| EU590825 | 2005 | South Korea | GenBank | C2 |
| EU590826 | 2005 | South Korea | GenBank | C2 |
| EU590827 | 2005 | South Korea | GenBank | C2 |
| EU590828 | 2005 | South Korea | GenBank | C2 |
| EU604664 | 2005 | South Korea | GenBank | C2 |
| FJ525936 | 2008 | UK | GenBank | C2 |
| FJ525940 | 2008 | UK | GenBank | C2 |
| FJ868306 | 2005 | Australia | GenBank | C2 |
| FJ868307 | 2005 | Australia | GenBank | C2 |
| FJ868347 | 2004 | Australia | GenBank | C2 |
| FJ868348 | 2005 | Australia | GenBank | C2 |
| FJ868349 | 2005 | Australia | GenBank | C2 |
| FJ868350 | 2005 | Australia | GenBank | C2 |
| FJ868351 | 2005 | Australia | GenBank | C2 |
| FJ868352 | 2005 | Australia | GenBank | C2 |
| FJ868353 | 2005 | Australia | GenBank | C2 |
| FJ868354 | 2005 | Australia | GenBank | C2 |
| GQ205563 | 2006 | China | GenBank | C2 |
| GQ205564 | 2006 | China | GenBank | C2 |
| GQ205565 | 2006 | China | GenBank | C2 |
| GQ205566 | 2006 | China | GenBank | C2 |
| GQ205567 | 2006 | China | GenBank | C2 |
| GQ205568 | 2006 | China | GenBank | C2 |
| GQ205569 | 2006 | China | GenBank | C2 |
| GQ205570 | 2006 | China | GenBank | C2 |
| GQ205571 | 2006 | China | GenBank | C2 |
| GQ205572 | 2006 | China | GenBank | C2 |
| GQ205573 | 2006 | China | GenBank | C2 |
| GQ205574 | 2006 | China | GenBank | C2 |
| GQ205575 | 2006 | China | GenBank | C2 |
| GQ205576 | 2006 | China | GenBank | C2 |
| GQ205577 | 2006 | China | GenBank | C2 |
| GQ205578 | 2006 | China | GenBank | C2 |
| GQ205579 | 2006 | China | GenBank | C2 |
| GQ222197 | 2007 | Greece | GenBank | C2 |
| GQ329813 | 2005 | China | GenBank | A |
| GU142897 | 2005 | Australia | GenBank | C2 |
| GU142898 | 2000 | Australia | GenBank | C1 |
| GU142899 | 2001 | Australia | GenBank | C1 |
| GU142900 | 2001 | Australia | GenBank | C1 |
| GU142901 | 2001 | Australia | GenBank | C1 |
| GU142902 | 1997 | Australia | GenBank | C2 |
| HF948104 | 2000 | France | GenBank | C1 |
| HF948105 | 2000 | France | GenBank | C1 |
| HF948106 | 2002 | France | GenBank | C1 |
| HF948107 | 2006 | France | GenBank | C1 |
| HF948108 | 2006 | France | GenBank | C1 |
| HG793717 | 2012 | France | GenBank | C2 |
| HG793718 | 2010 | France | GenBank | C2 |
| HG793719 | 2011 | France | GenBank | C1 |
| HG793720 | 2011 | France | GenBank | C2 |
| HG793721 | 2011 | France | GenBank | C1 |
| HG793722 | 2011 | France | GenBank | C1 |
| HG793723 | 2011 | France | GenBank | C1 |
| HG793724 | 2012 | France | GenBank | C1 |
| HG793725 | 2012 | France | GenBank | C2 |
| HG793726 | 2012 | France | GenBank | C2 |
| HG793727 | 2012 | France | GenBank | C2 |
| HM777023 | 2005 | South Korea | GenBank | C2 |
| JF896202 | 2011 | India | GenBank | B |
| JN034223 | 2002 | Germany | GenBank | B |
| JN203849 | 2011 | India | GenBank | B |
| JN203850 | 2011 | India | GenBank | B |
| JN203851 | 2011 | India | GenBank | B |
| JN203852 | 2011 | India | GenBank | B |
| JQ239030 | 2006 | Sweden | GenBank | C2 |
| JX139826 | 2010 | Russia | GenBank | C1 |
| JX473479 | 2011 | China | GenBank | A |
| JX476244 | 2009 | India | GenBank | B |
| JX476245 | 2009 | India | GenBank | B |
| JX538068 | 2007 | Bangladesh | GenBank | B |
| JX538069 | 2007 | Bangladesh | GenBank | B |
| KC893478 | 2009 | Netherlands | GenBank | C2 |
| KC893479 | 2009 | Netherlands | GenBank | C2 |
| KC893482 | 2009 | Netherlands | GenBank | C2 |
| KC893504 | 2010 | Netherlands | GenBank | C2 |
| KC893506 | 2010 | Netherlands | GenBank | C2 |
| KF412979 | 2008 | India | GenBank | B |
| KF412994 | 2008 | India | GenBank | B |
| KF413061 | 2009 | India | GenBank | B |
| KJ632589 | 2010 | India | GenBank | B |
| KJ649261 | 2013 | Italy | GenBank | C2 |
| KJ746497 | 2014 | Italy | GenBank | C2 |
| KM820892 | 2014 | Italy | GenBank | C1 |
| KT364218 | 2014 | Italy | GenBank | C1 |
| KT693321 | 2014 | Russia | GenBank | C1 |
| KT693322 | 2014 | Russia | GenBank | C1 |
| KT693323 | 2014 | Russia | GenBank | C1 |
| KT693324 | 2014 | Russia | GenBank | C1 |
| KT693325 | 2014 | Russia | GenBank | C1 |
| KT693326 | 2014 | Russia | GenBank | C1 |
| KT693327 | 2014 | Russia | GenBank | C1 |
| KT693328 | 2014 | Russia | GenBank | C1 |
| KT693329 | 2014 | Russia | GenBank | C1 |
| KT693330 | 2014 | Russia | GenBank | C1 |
| KT693331 | 2014 | Russia | GenBank | C1 |
| KT693332 | 2014 | Russia | GenBank | C1 |
| KT853019 | 2014 | Italy | GenBank | C1 |
| KU133587 | 2012 | Russia | GenBank | C1 |
| KU133609 | 2012 | Russia | GenBank | C1 |
| KU133621 | 2013 | Russia | GenBank | C1 |
| KU133628 | 2013 | Russia | GenBank | C1 |
| KU216191 | 2015 | China | GenBank | C2 |
| KU216192 | 2015 | China | GenBank | C2 |
| KU216193 | 2015 | China | GenBank | C2 |
| KU216194 | 2015 | China | GenBank | C2 |
| KU216195 | 2015 | China | GenBank | C2 |
| KU216196 | 2015 | China | GenBank | C2 |
| KU216197 | 2015 | China | GenBank | C2 |
| KU216198 | 2015 | China | GenBank | C2 |
| KU216199 | 2015 | China | GenBank | C2 |
| KU216200 | 2015 | China | GenBank | C2 |
| KU216201 | 2015 | China | GenBank | C2 |
| KU216202 | 2015 | China | GenBank | C2 |
| KU216203 | 2015 | China | GenBank | C2 |
| KU216204 | 2015 | China | GenBank | C2 |
| KU216205 | 2015 | China | GenBank | C2 |
| KU297237 | 2014 | Italy | GenBank | C1 |
| KU555717 | 2014 | Italy | GenBank | C1 |
| KU561036 | 2011 | Netherlands | GenBank | C2 |
| KU561037 | 2011 | Netherlands | GenBank | C2 |
| KU561038 | 2011 | Netherlands | GenBank | C2 |
| KU561039 | 2011 | Netherlands | GenBank | C2 |
| KU561040 | 2011 | Netherlands | GenBank | C2 |
| KU561041 | 2011 | Netherlands | GenBank | C2 |
| KU561042 | 2011 | Netherlands | GenBank | C2 |
| KU574621 | 2010 | Thailand | GenBank | C2 |
| KX139439 | 2010 | Germany | GenBank | C1 |
| KX139441 | 2010 | Germany | GenBank | C1 |
| KX139444 | 2010 | Germany | GenBank | C1 |
| KX139446 | 2010 | Germany | GenBank | C1 |
| KX139447 | 2010 | Germany | GenBank | C1 |
| KX139448 | 2010 | Germany | GenBank | C1 |
| KX139449 | 2010 | Germany | GenBank | C1 |
| KX139450 | 2010 | Germany | GenBank | C1 |
| KX139451 | 2010 | Germany | GenBank | C1 |
| KX139452 | 2010 | Germany | GenBank | C1 |
| KX139453 | 2010 | Germany | GenBank | C1 |
| KX139454 | 2010 | Germany | GenBank | C1 |
| KX139455 | 2010 | Germany | GenBank | C1 |
| KX139456 | 2010 | Germany | GenBank | C1 |
| KX139457 | 2010 | Germany | GenBank | C1 |
| KX139458 | 2010 | Germany | GenBank | C1 |
| KX767786 | 2015 | China | GenBank | C2 |
| KY303773 | 2015 | China | GenBank | C2 |
| KY303774 | 2015 | China | GenBank | C2 |
| KY303775 | 2015 | China | GenBank | C2 |
| KY303776 | 2015 | China | GenBank | C2 |
| KY303777 | 2015 | China | GenBank | C2 |
| KY303778 | 2015 | China | GenBank | C2 |
| KY303779 | 2015 | China | GenBank | C2 |
| KY303780 | 2015 | China | GenBank | C2 |
| KY303781 | 2015 | China | GenBank | C2 |
| KY303782 | 2015 | China | GenBank | C2 |
| KY303783 | 2015 | China | GenBank | C2 |
| KY303784 | 2015 | China | GenBank | C2 |
| KY303785 | 2015 | China | GenBank | C2 |
| KY303786 | 2015 | China | GenBank | C2 |
| KY303787 | 2015 | China | GenBank | C2 |
| KY303788 | 2015 | China | GenBank | C2 |
| KY303789 | 2015 | China | GenBank | C2 |
| KY303790 | 2015 | China | GenBank | C2 |
| KY303791 | 2015 | China | GenBank | C2 |
| KY303792 | 2015 | China | GenBank | C2 |
| KY303793 | 2015 | China | GenBank | C2 |
| KY303794 | 2015 | China | GenBank | C2 |
| KY303795 | 2015 | China | GenBank | C2 |
| KY303796 | 2015 | China | GenBank | C2 |
| KY303797 | 2015 | China | GenBank | C2 |
| KY303798 | 2015 | China | GenBank | C2 |
| KY303799 | 2015 | China | GenBank | C2 |
| KY303800 | 2015 | China | GenBank | C2 |
| KY303801 | 2015 | China | GenBank | C2 |
| KY303802 | 2015 | China | GenBank | C2 |
| KY303803 | 2015 | China | GenBank | C2 |
| KY303804 | 2015 | China | GenBank | C2 |
| KY303805 | 2015 | China | GenBank | C2 |
| KY303806 | 2015 | China | GenBank | C2 |
| KY303807 | 2015 | China | GenBank | C2 |
| KY303808 | 2015 | China | GenBank | C2 |
| KY303809 | 2015 | China | GenBank | C2 |
| KY303810 | 2015 | China | GenBank | C2 |
| KY303811 | 2015 | China | GenBank | C2 |
| KY303812 | 2015 | China | GenBank | C2 |
| KY303813 | 2015 | China | GenBank | C2 |
| KY303814 | 2015 | China | GenBank | C2 |
| KY303815 | 2015 | China | GenBank | C2 |
| KY303816 | 2015 | China | GenBank | C2 |
| KY303817 | 2015 | China | GenBank | C2 |
| KY303818 | 2015 | China | GenBank | C2 |
| KY303819 | 2015 | China | GenBank | C2 |
| KY303820 | 2015 | China | GenBank | C2 |
| KY303821 | 2015 | China | GenBank | C2 |
| KY303822 | 2015 | China | GenBank | C2 |
| KY303823 | 2015 | China | GenBank | C2 |
| KY303824 | 2015 | China | GenBank | C2 |
| KY303825 | 2015 | China | GenBank | C2 |
| KY303826 | 2015 | China | GenBank | C2 |
| KY303827 | 2015 | China | GenBank | C2 |
| KY303828 | 2015 | China | GenBank | C2 |
| KY303829 | 2015 | China | GenBank | C2 |
| KY433734 | 2014 | Niger | GenBank | B |
| KY433735 | 2014 | Mauritania | GenBank | B |
| KY433736 | 2014 | Niger | GenBank | B |
| KY828851 | 2016 | China | GenBank | C2 |
| KY828852 | 2016 | China | GenBank | C2 |
| KY865770 | 2012 | Netherlands | GenBank | C1 |
| KY865780 | 2013 | Netherlands | GenBank | C1 |
| KY866103 | 2014 | Netherlands | GenBank | C2 |
| KY866125 | 2014 | Netherlands | GenBank | C2 |
| KY866294 | 2014 | Netherlands | GenBank | C2 |
| KY866335 | 2014 | Netherlands | GenBank | C2 |
| KY866384 | 2014 | Netherlands | GenBank | C2 |
| KY866457 | 2014 | Netherlands | GenBank | C2 |
| KY866470 | 2014 | Netherlands | GenBank | C2 |
| KY866476 | 2014 | Netherlands | GenBank | C2 |
| KY866489 | 2014 | Netherlands | GenBank | C2 |
| KY866522 | 2014 | Netherlands | GenBank | C2 |
| KY866568 | 2014 | Netherlands | GenBank | C2 |
| KY866573 | 2014 | Netherlands | GenBank | C2 |
| KY866583 | 2014 | Netherlands | GenBank | C2 |
| KY866598 | 2014 | Netherlands | GenBank | C2 |
| KY866601 | 2014 | Netherlands | GenBank | C2 |
| KY866635 | 2015 | Netherlands | GenBank | C2 |
| LC106305 | 2015 | Japan | GenBank | C2 |
| LC106306 | 2015 | Japan | GenBank | C2 |
| LC383283 | 2015 | Japan | GenBank | C2 |
| LC707444 | 2021 | China | GenBank | C2 |
| LC707449 | 2021 | China | GenBank | C2 |
| LC707464 | 2021 | China | GenBank | C2 |
| LN713457 | 2012 | Tunisia | GenBank | C2 |
| LT883145 | 2017 | Tunisia | GenBank | C2 |
| MF160175 | 2014 | USA | GenBank | C2 |
| MF160210 | 2014 | USA | GenBank | C2 |
| MF160229 | 2014 | USA | GenBank | C2 |
| MF160232 | 2014 | USA | GenBank | C2 |
| MF160242 | 2014 | USA | GenBank | C2 |
| MF467308 | 2014 | China | GenBank | C2 |
| MF467309 | 2014 | China | GenBank | C2 |
| MF467310 | 2014 | China | GenBank | C2 |
| MF467311 | 2014 | China | GenBank | C2 |
| MF467312 | 2015 | China | GenBank | C2 |
| MF467313 | 2015 | China | GenBank | C2 |
| MF467314 | 2016 | China | GenBank | C2 |
| MF589254 | 2014 | Japan | GenBank | C2 |
| MF589290 | 2015 | Japan | GenBank | C2 |
| MF589298 | 2016 | Japan | GenBank | C2 |
| MF678301 | 2008 | Australia | GenBank | C2 |
| MF838733 | 2011 | Australia | GenBank | C2 |
| MF990301 | 2016 | Ethiopia | GenBank | B |
| MG720242 | 2016 | China | GenBank | C2 |
| MG720243 | 2015 | China | GenBank | C2 |
| MG720244 | 2015 | China | GenBank | C2 |
| MG720245 | 2015 | China | GenBank | C2 |
| MG720246 | 2015 | China | GenBank | C2 |
| MG720247 | 2015 | China | GenBank | C2 |
| MG720248 | 2015 | China | GenBank | C2 |
| MG720249 | 2015 | China | GenBank | C2 |
| MG720250 | 2015 | China | GenBank | C2 |
| MG720251 | 2015 | China | GenBank | C2 |
| MG720252 | 2015 | China | GenBank | C2 |
| MG720253 | 2015 | China | GenBank | C2 |
| MG720254 | 2015 | China | GenBank | C2 |
| MG720255 | 2015 | China | GenBank | C2 |
| MG720256 | 2015 | China | GenBank | C2 |
| MG720257 | 2015 | China | GenBank | C2 |
| MG720258 | 2015 | China | GenBank | C2 |
| MG720259 | 2015 | China | GenBank | C2 |
| MG720260 | 2015 | China | GenBank | C2 |
| MG720261 | 2015 | China | GenBank | C2 |
| MG773498 | 2017 | West Bank | GenBank | C2 |
| MG773499 | 2017 | West Bank | GenBank | C2 |
| MG773500 | 2017 | West Bank | GenBank | C2 |
| MG773501 | 2017 | West Bank | GenBank | C2 |
| MG773502 | 2017 | West Bank | GenBank | C2 |
| MG773503 | 2017 | West Bank | GenBank | C2 |
| MG773504 | 2017 | West Bank | GenBank | C2 |
| MG773505 | 2017 | West Bank | GenBank | C2 |
| MG773506 | 2017 | West Bank | GenBank | C2 |
| MG773507 | 2017 | West Bank | GenBank | C2 |
| MG773508 | 2017 | West Bank | GenBank | C2 |
| MG773509 | 2017 | West Bank | GenBank | C2 |
| MG773510 | 2017 | West Bank | GenBank | C2 |
| MH021890 | 2008 | Vietnam | GenBank | C2 |
| MH118976 | 2017 | China | GenBank | C2 |
| MH118977 | 2017 | China | GenBank | C2 |
| MH118978 | 2017 | China | GenBank | C2 |
| MH118979 | 2017 | China | GenBank | C2 |
| MH310895 | 2015 | USA | GenBank | C2 |
| MH310899 | 2015 | USA | GenBank | C2 |
| MH310900 | 2015 | USA | GenBank | C2 |
| MH716181 | 2015 | China | GenBank | C2 |
| MK086375 | 2014 | France | GenBank | C2 |
| MK086376 | 2014 | France | GenBank | C2 |
| MK086377 | 2014 | France | GenBank | C2 |
| MK111295 | 2013 | Cyprus | GenBank | C2 |
| MK111296 | 2013 | Cyprus | GenBank | C2 |
| MK111297 | 2013 | Cyprus | GenBank | C2 |
| MK111298 | 2013 | Cyprus | GenBank | C2 |
| MK111299 | 2017 | Cyprus | GenBank | C2 |
| MK111300 | 2017 | Cyprus | GenBank | C2 |
| MK111301 | 2017 | Cyprus | GenBank | C2 |
| MK111302 | 2017 | Cyprus | GenBank | C2 |
| MK111303 | 2017 | Cyprus | GenBank | C2 |
| MK111304 | 2017 | Cyprus | GenBank | C2 |
| MK111305 | 2017 | Cyprus | GenBank | C2 |
| MK256761 | 2015 | China | GenBank | C2 |
| MK256762 | 2015 | China | GenBank | C2 |
| MK307334 | 2018 | China | GenBank | C2 |
| MK307335 | 2018 | China | GenBank | C2 |
| MK307336 | 2018 | China | GenBank | C2 |
| MK307337 | 2018 | China | GenBank | C2 |
| MK307338 | 2018 | China | GenBank | C2 |
| MK307339 | 2018 | China | GenBank | C2 |
| MK307340 | 2018 | China | GenBank | C2 |
| MK307341 | 2018 | China | GenBank | C2 |
| MK307342 | 2018 | China | GenBank | C2 |
| MK307343 | 2018 | China | GenBank | C2 |
| MK531854 | 2017 | Nigeria | GenBank | B |
| MK531855 | 2017 | Nigeria | GenBank | B |
| MK836155 | 2018 | USA | GenBank | C2 |
| MK836172 | 2018 | USA | GenBank | C2 |
| MN052953 | 2017 | China | GenBank | C2 |
| MN052954 | 2014 | China | GenBank | C2 |
| MN166092 | 2015 | USA | GenBank | C2 |
| MN215884 | 2019 | China | GenBank | C2 |
| MN337405 | 2019 | China | GenBank | C2 |
| MN541049 | 2018 | China | GenBank | C2 |
| MN541053 | 2018 | China | GenBank | C2 |
| MN688218 | 2019 | China | GenBank | C2 |
| MN737181 | 2019 | China | GenBank | C2 |
| MN737182 | 2019 | China | GenBank | C2 |
| MN737183 | 2019 | China | GenBank | C2 |
| MN737184 | 2019 | China | GenBank | C2 |
| MN737185 | 2019 | China | GenBank | C2 |
| MN737186 | 2019 | China | GenBank | C2 |
| MN737187 | 2019 | China | GenBank | C2 |
| MN737188 | 2019 | China | GenBank | C2 |
| MN737189 | 2019 | China | GenBank | C2 |
| MN737190 | 2019 | China | GenBank | C2 |
| MN749143 | 2015 | USA | GenBank | C2 |
| MN749146 | 2015 | USA | GenBank | C2 |
| MN792654 | 2019 | China | GenBank | C2 |
| MN808792 | 2019 | China | GenBank | C2 |
| MN808793 | 2019 | China | GenBank | C2 |
| MN808794 | 2019 | China | GenBank | C2 |
| MN815810 | 2018 | China | GenBank | C2 |
| MN815811 | 2018 | China | GenBank | C2 |
| MN815812 | 2019 | China | GenBank | C2 |
| MN815813 | 2018 | China | GenBank | C2 |
| MN832717 | 2019 | China | GenBank | C2 |
| MN832718 | 2019 | China | GenBank | C2 |
| MN896908 | 2019 | USA | GenBank | C2 |
| MN896909 | 2019 | USA | GenBank | C2 |
| MN896910 | 2019 | USA | GenBank | C2 |
| MN896914 | 2019 | USA | GenBank | C2 |
| MT350224 | 2019 | China | GenBank | C2 |
| MT491215 | 2010 | Brazil | GenBank | C2 |
| MT614259 | 2014 | UK | GenBank | C2 |
| MT641376 | 2017 | UK | GenBank | C2 |
| MT641383 | 2017 | UK | GenBank | C2 |
| MT641413 | 2018 | UK | GenBank | C2 |
| MT641418 | 2018 | UK | GenBank | C2 |
| MT755385 | 2019 | China | GenBank | C2 |
| MT950544 | 2019 | China | GenBank | C2 |
| MT950546 | 2019 | China | GenBank | C2 |
| MT950549 | 2019 | China | GenBank | C2 |
| MT950550 | 2019 | China | GenBank | C2 |
| MT950552 | 2019 | China | GenBank | C2 |
| MT950553 | 2019 | China | GenBank | C2 |
| MT950556 | 2019 | China | GenBank | C2 |
| MT950558 | 2019 | China | GenBank | C2 |
| MT950559 | 2019 | China | GenBank | C2 |
| MT950562 | 2019 | China | GenBank | C2 |
| MT950563 | 2019 | China | GenBank | C2 |
| MT950566 | 2019 | China | GenBank | C2 |
| MT950568 | 2019 | China | GenBank | C2 |
| MT950570 | 2019 | China | GenBank | C2 |
| MT950571 | 2019 | China | GenBank | C2 |
| MT950572 | 2019 | China | GenBank | C2 |
| MT950573 | 2019 | China | GenBank | C2 |
| MT950574 | 2019 | China | GenBank | C2 |
| MT950576 | 2019 | China | GenBank | C2 |
| MT950577 | 2019 | China | GenBank | C2 |
| MT950578 | 2019 | China | GenBank | C2 |
| MT950580 | 2019 | China | GenBank | C2 |
| MT950581 | 2019 | China | GenBank | C2 |
| MT950582 | 2019 | China | GenBank | C2 |
| MT950583 | 2019 | China | GenBank | C2 |
| MT950584 | 2019 | China | GenBank | C2 |
| MT950585 | 2019 | China | GenBank | C2 |
| MT950586 | 2019 | China | GenBank | C2 |
| MT950587 | 2019 | China | GenBank | C2 |
| MT950588 | 2019 | China | GenBank | C2 |
| MT950590 | 2019 | China | GenBank | C2 |
| MT950592 | 2019 | China | GenBank | C2 |
| MT950593 | 2019 | China | GenBank | C2 |
| MT950594 | 2019 | China | GenBank | C2 |
| MT950595 | 2019 | China | GenBank | C2 |
| MT950596 | 2019 | China | GenBank | C2 |
| MT950597 | 2019 | China | GenBank | C2 |
| MT950598 | 2019 | China | GenBank | C2 |
| MT950626 | 2018 | China | GenBank | C2 |
| MT950627 | 2018 | China | GenBank | C2 |
| MT950628 | 2018 | China | GenBank | C2 |
| MT950629 | 2018 | China | GenBank | C2 |
| MT950630 | 2018 | China | GenBank | C2 |
| MT950631 | 2018 | China | GenBank | C2 |
| MT950632 | 2018 | China | GenBank | C2 |
| MT950633 | 2018 | China | GenBank | C2 |
| MT950634 | 2018 | China | GenBank | C2 |
| MT950635 | 2018 | China | GenBank | C2 |
| MT950636 | 2018 | China | GenBank | C2 |
| MW481634 | 2019 | China | GenBank | C2 |
| MW731975 | 2014 | Germany | GenBank | C2 |
| MW731976 | 2014 | Germany | GenBank | C2 |
| MW731977 | 2015 | Germany | GenBank | C2 |
| MW731978 | 2015 | Germany | GenBank | C2 |
| MW731979 | 2015 | Germany | GenBank | C2 |
| MW731980 | 2016 | Germany | GenBank | C2 |
| MW731981 | 2016 | Germany | GenBank | C2 |
| OK585080 | 2019 | Mayotte | GenBank | C2 |
| ON985268 | 2020 | India | GenBank | C2 |
| ON985269 | 2020 | India | GenBank | C2 |
| OQ791557 | 2015 | USA | GenBank | C2 |
| OQ791558 | 2015 | USA | GenBank | C2 |
| OQ791559 | 2015 | USA | GenBank | C2 |
| OQ791562 | 2015 | USA | GenBank | C2 |
| OQ791564 | 2015 | USA | GenBank | C2 |
| OQ842421 | 2012 | USA | GenBank | C1 |
| OQ842422 | 2012 | USA | GenBank | C1 |
| AF081331 | 1955 | USA | GenBank | Metcalf |
| AF317694 | 2000 | Sweden | GenBank | Metcalf |

USA: United States of America; UK: United Kingdom.
