# Supplementary material for: Circulated echovirus 18 strains in Guangdong Province and worldwide: A novel perspective on genetic diversity and recombination patterns
Source: Virulence. 2025 Jul 15;16(1):2534519. doi: 10.1080/21505594.2025.2534519 (PMC12296116; doi:10.1080/21505594.2025.2534519)
Supplement: Supplemental Material [file KVIR_A_2534519_SM5328.zip › Supplementary File_1_Table_S3.docx]

**Supplementary Table S3.** Geographic distribution of information on 229 E18 strains in China.

| Accession | Source | Province | Year |
| --- | --- | --- | --- |
| PP891437 | This study | Guangdong | 2019 |
| PP891438 | This study | Guangdong | 2019 |
| PP891439 | This study | Guangdong | 2019 |
| PP891440 | This study | Guangdong | 2019 |
| PP891441 | This study | Guangdong | 2022 |
| PP891442 | This study | Guangdong | 2022 |
| PP891443 | This study | Guangdong | 2022 |
| AB268227 | GenBank | Yunnan | 2000 |
| AB268228 | GenBank | Yunnan | 2000 |
| GQ205563 | GenBank | Taiwan | 2006 |
| GQ205564 | GenBank | Taiwan | 2006 |
| GQ205565 | GenBank | Taiwan | 2006 |
| GQ205566 | GenBank | Taiwan | 2006 |
| GQ205567 | GenBank | Taiwan | 2006 |
| GQ205568 | GenBank | Taiwan | 2006 |
| GQ205569 | GenBank | Taiwan | 2006 |
| GQ205570 | GenBank | Taiwan | 2006 |
| GQ205571 | GenBank | Taiwan | 2006 |
| GQ205572 | GenBank | Taiwan | 2006 |
| GQ205573 | GenBank | Taiwan | 2006 |
| GQ205574 | GenBank | Taiwan | 2006 |
| GQ205575 | GenBank | Taiwan | 2006 |
| GQ205576 | GenBank | Taiwan | 2006 |
| GQ205577 | GenBank | Taiwan | 2006 |
| GQ205578 | GenBank | Taiwan | 2006 |
| GQ205579 | GenBank | Taiwan | 2006 |
| GQ329813 | GenBank | Shandong | 2005 |
| JX473479 | GenBank | Guangdong | 2011 |
| KU216191 | GenBank | Hebei | 2015 |
| KU216192 | GenBank | Hebei | 2015 |
| KU216193 | GenBank | Hebei | 2015 |
| KU216194 | GenBank | Hebei | 2015 |
| KU216195 | GenBank | Hebei | 2015 |
| KU216196 | GenBank | Hebei | 2015 |
| KU216197 | GenBank | Hebei | 2015 |
| KU216198 | GenBank | Hebei | 2015 |
| KU216199 | GenBank | Hebei | 2015 |
| KU216200 | GenBank | Hebei | 2015 |
| KU216201 | GenBank | Hebei | 2015 |
| KU216202 | GenBank | Hebei | 2015 |
| KU216203 | GenBank | Hebei | 2015 |
| KU216204 | GenBank | Hebei | 2015 |
| KU216205 | GenBank | Hebei | 2015 |
| KX767786 | GenBank | Hebei | 2015 |
| KY303773 | GenBank | Hebei | 2015 |
| KY303774 | GenBank | Hebei | 2015 |
| KY303775 | GenBank | Hebei | 2015 |
| KY303776 | GenBank | Hebei | 2015 |
| KY303777 | GenBank | Hebei | 2015 |
| KY303778 | GenBank | Hebei | 2015 |
| KY303779 | GenBank | Hebei | 2015 |
| KY303780 | GenBank | Hebei | 2015 |
| KY303781 | GenBank | Hebei | 2015 |
| KY303782 | GenBank | Hebei | 2015 |
| KY303783 | GenBank | Hebei | 2015 |
| KY303784 | GenBank | Hebei | 2015 |
| KY303785 | GenBank | Hebei | 2015 |
| KY303786 | GenBank | Hebei | 2015 |
| KY303787 | GenBank | Hebei | 2015 |
| KY303788 | GenBank | Hebei | 2015 |
| KY303789 | GenBank | Hebei | 2015 |
| KY303790 | GenBank | Hebei | 2015 |
| KY303791 | GenBank | Hebei | 2015 |
| KY303792 | GenBank | Hebei | 2015 |
| KY303793 | GenBank | Hebei | 2015 |
| KY303794 | GenBank | Hebei | 2015 |
| KY303795 | GenBank | Hebei | 2015 |
| KY303796 | GenBank | Hebei | 2015 |
| KY303797 | GenBank | Hebei | 2015 |
| KY303798 | GenBank | Hebei | 2015 |
| KY303799 | GenBank | Hebei | 2015 |
| KY303800 | GenBank | Hebei | 2015 |
| KY303801 | GenBank | Hebei | 2015 |
| KY303802 | GenBank | Hebei | 2015 |
| KY303803 | GenBank | Hebei | 2015 |
| KY303804 | GenBank | Hebei | 2015 |
| KY303805 | GenBank | Hebei | 2015 |
| KY303806 | GenBank | Hebei | 2015 |
| KY303807 | GenBank | Hebei | 2015 |
| KY303808 | GenBank | Hebei | 2015 |
| KY303809 | GenBank | Hebei | 2015 |
| KY303810 | GenBank | Hebei | 2015 |
| KY303811 | GenBank | Hebei | 2015 |
| KY303812 | GenBank | Hebei | 2015 |
| KY303813 | GenBank | Hebei | 2015 |
| KY303814 | GenBank | Hebei | 2015 |
| KY303815 | GenBank | Hebei | 2015 |
| KY303816 | GenBank | Hebei | 2015 |
| KY303817 | GenBank | Hebei | 2015 |
| KY303818 | GenBank | Hebei | 2015 |
| KY303819 | GenBank | Hebei | 2015 |
| KY303820 | GenBank | Hebei | 2015 |
| KY303821 | GenBank | Hebei | 2015 |
| KY303822 | GenBank | Hebei | 2015 |
| KY303823 | GenBank | Hebei | 2015 |
| KY303824 | GenBank | Hebei | 2015 |
| KY303825 | GenBank | Hebei | 2015 |
| KY303826 | GenBank | Hebei | 2015 |
| KY303827 | GenBank | Hebei | 2015 |
| KY303828 | GenBank | Hebei | 2015 |
| KY303829 | GenBank | Hebei | 2015 |
| KY828851 | GenBank | Yunnan | 2016 |
| KY828852 | GenBank | Yunnan | 2016 |
| LC707444 | GenBank | Yunnan | 2021 |
| LC707449 | GenBank | Yunnan | 2021 |
| LC707464 | GenBank | Yunnan | 2021 |
| MF467308 | GenBank | Zhejiang | 2014 |
| MF467309 | GenBank | Zhejiang | 2014 |
| MF467310 | GenBank | Zhejiang | 2014 |
| MF467311 | GenBank | Zhejiang | 2014 |
| MF467312 | GenBank | Zhejiang | 2015 |
| MF467313 | GenBank | Zhejiang | 2015 |
| MF467314 | GenBank | Zhejiang | 2016 |
| MG720242 | GenBank | Yunnan | 2016 |
| MG720243 | GenBank | Hebei | 2015 |
| MG720244 | GenBank | Hebei | 2015 |
| MG720245 | GenBank | Heilongjiang | 2015 |
| MG720246 | GenBank | Heilongjiang | 2015 |
| MG720247 | GenBank | Heilongjiang | 2015 |
| MG720248 | GenBank | Heilongjiang | 2015 |
| MG720249 | GenBank | Jiangsu | 2015 |
| MG720250 | GenBank | Jiangsu | 2015 |
| MG720251 | GenBank | Jiangsu | 2015 |
| MG720252 | GenBank | Jiangsu | 2015 |
| MG720253 | GenBank | Shaanxi | 2015 |
| MG720254 | GenBank | Shandong | 2015 |
| MG720255 | GenBank | Shandong | 2015 |
| MG720256 | GenBank | Hebei | 2015 |
| MG720257 | GenBank | Hebei | 2015 |
| MG720258 | GenBank | Hebei | 2015 |
| MG720259 | GenBank | Hebei | 2015 |
| MG720260 | GenBank | Hebei | 2015 |
| MG720261 | GenBank | Hebei | 2015 |
| MH118976 | GenBank | Zhejiang | 2017 |
| MH118977 | GenBank | Zhejiang | 2017 |
| MH118978 | GenBank | Zhejiang | 2017 |
| MH118979 | GenBank | Zhejiang | 2017 |
| MH716181 | GenBank | Guangdong | 2015 |
| MK256761 | GenBank | Hebei | 2015 |
| MK256762 | GenBank | Hebei | 2015 |
| MK307334 | GenBank | Hunan | 2018 |
| MK307335 | GenBank | Hunan | 2018 |
| MK307336 | GenBank | Hunan | 2018 |
| MK307337 | GenBank | Hunan | 2018 |
| MK307338 | GenBank | Hunan | 2018 |
| MK307339 | GenBank | Hunan | 2018 |
| MK307340 | GenBank | Hunan | 2018 |
| MK307341 | GenBank | Hunan | 2018 |
| MK307342 | GenBank | Hunan | 2018 |
| MK307343 | GenBank | Hunan | 2018 |
| MN052953 | GenBank | Zhejiang | 2017 |
| MN052954 | GenBank | Zhejiang | 2014 |
| MN215884 | GenBank | Guangdong | 2019 |
| MN337405 | GenBank | Guangdong | 2019 |
| MN541049 | GenBank | Shandong | 2018 |
| MN541053 | GenBank | Shandong | 2018 |
| MN688218 | GenBank | Guangdong | 2019 |
| MN737181 | GenBank | Shandong | 2019 |
| MN737182 | GenBank | Shandong | 2019 |
| MN737183 | GenBank | Shandong | 2019 |
| MN737184 | GenBank | Shandong | 2019 |
| MN737185 | GenBank | Shandong | 2019 |
| MN737186 | GenBank | Shandong | 2019 |
| MN737187 | GenBank | Shandong | 2019 |
| MN737188 | GenBank | Shandong | 2019 |
| MN737189 | GenBank | Shandong | 2019 |
| MN737190 | GenBank | Shandong | 2019 |
| MN792654 | GenBank | Guangdong | 2019 |
| MN808792 | GenBank | Hebei | 2019 |
| MN808793 | GenBank | Hebei | 2019 |
| MN808794 | GenBank | Guangdong | 2019 |
| MN815810 | GenBank | Hebei | 2018 |
| MN815811 | GenBank | Hebei | 2018 |
| MN815812 | GenBank | Hebei | 2019 |
| MN815813 | GenBank | Hebei | 2018 |
| MN832717 | GenBank | Shandong | 2019 |
| MN832718 | GenBank | Shandong | 2019 |
| MT350224 | GenBank | Jiangxi | 2019 |
| MT755385 | GenBank | Jiangsu | 2019 |
| MT950544 | GenBank | Shandong | 2019 |
| MT950546 | GenBank | Shandong | 2019 |
| MT950549 | GenBank | Shandong | 2019 |
| MT950550 | GenBank | Shandong | 2019 |
| MT950552 | GenBank | Shandong | 2019 |
| MT950553 | GenBank | Shandong | 2019 |
| MT950556 | GenBank | Shandong | 2019 |
| MT950558 | GenBank | Shandong | 2019 |
| MT950559 | GenBank | Shandong | 2019 |
| MT950562 | GenBank | Shandong | 2019 |
| MT950563 | GenBank | Shandong | 2019 |
| MT950566 | GenBank | Shandong | 2019 |
| MT950568 | GenBank | Shandong | 2019 |
| MT950570 | GenBank | Shandong | 2019 |
| MT950571 | GenBank | Shandong | 2019 |
| MT950572 | GenBank | Shandong | 2019 |
| MT950573 | GenBank | Shandong | 2019 |
| MT950574 | GenBank | Shandong | 2019 |
| MT950576 | GenBank | Shandong | 2019 |
| MT950577 | GenBank | Shandong | 2019 |
| MT950578 | GenBank | Shandong | 2019 |
| MT950580 | GenBank | Shandong | 2019 |
| MT950581 | GenBank | Shandong | 2019 |
| MT950582 | GenBank | Shandong | 2019 |
| MT950583 | GenBank | Shandong | 2019 |
| MT950584 | GenBank | Shandong | 2019 |
| MT950585 | GenBank | Shandong | 2019 |
| MT950586 | GenBank | Shandong | 2019 |
| MT950587 | GenBank | Shandong | 2019 |
| MT950588 | GenBank | Shandong | 2019 |
| MT950590 | GenBank | Shandong | 2019 |
| MT950592 | GenBank | Shandong | 2019 |
| MT950593 | GenBank | Shandong | 2019 |
| MT950594 | GenBank | Shandong | 2019 |
| MT950595 | GenBank | Shandong | 2019 |
| MT950596 | GenBank | Shandong | 2019 |
| MT950597 | GenBank | Shandong | 2019 |
| MT950598 | GenBank | Shandong | 2019 |
| MT950626 | GenBank | Shandong | 2018 |
| MT950627 | GenBank | Shandong | 2018 |
| MT950628 | GenBank | Shandong | 2018 |
| MT950629 | GenBank | Shandong | 2018 |
| MT950630 | GenBank | Shandong | 2018 |
| MT950631 | GenBank | Shandong | 2018 |
| MT950632 | GenBank | Shandong | 2018 |
| MT950633 | GenBank | Shandong | 2018 |
| MT950634 | GenBank | Shandong | 2018 |
| MT950635 | GenBank | Shandong | 2018 |
| MT950636 | GenBank | Shandong | 2018 |
| MW481634 | GenBank | Yunnan | 2019 |
