# Supplementary material for: Circulated echovirus 18 strains in Guangdong Province and worldwide: A novel perspective on genetic diversity and recombination patterns
Source: Virulence. 2025 Jul 15;16(1):2534519. doi: 10.1080/21505594.2025.2534519 (PMC12296116; doi:10.1080/21505594.2025.2534519)
Supplement: Supplemental Material [file KVIR_A_2534519_SM5328.zip › Supplementary File_1_Table_S4.docx]

**Supplementary Table S4.** Information on 342 sequences of E18 for the analysis of temporal distribution and seasonality characteristics.

| Accession | Month | Source | Genotype |
| --- | --- | --- | --- |
| PP891437 | Jun | This study | C2 |
| PP891438 | Jun | This study | C2 |
| PP891439 | Jun | This study | C2 |
| PP891440 | Aug | This study | C2 |
| PP891441 | May | This study | C2 |
| PP891442 | May | This study | C2 |
| PP891443 | Jun | This study | C2 |
| AB698775 | Jan | GenBank | C1 |
| AB902836 | Aug | GenBank | C2 |
| AB920409 | Jan | GenBank | C2 |
| AM236918 | May | GenBank | C2 |
| AM236953 | Sep | GenBank | C2 |
| AM236956 | Oct | GenBank | C2 |
| AM236957 | Oct | GenBank | C1 |
| AM236970 | Apr | GenBank | C1 |
| AM236972 | May | GenBank | C1 |
| AM236978 | Jun | GenBank | C1 |
| AM236984 | Jun | GenBank | C2 |
| AY342659 | May | GenBank | C1 |
| AY342660 | May | GenBank | C1 |
| AY342663 | Feb | GenBank | C1 |
| AY342673 | Apr | GenBank | C1 |
| AY342675 | Apr | GenBank | C1 |
| AY342676 | Mar | GenBank | C1 |
| AY342677 | Mar | GenBank | C1 |
| AY342678 | Mar | GenBank | C1 |
| AY342679 | Mar | GenBank | C1 |
| AY342688 | Mar | GenBank | C1 |
| AY342689 | Mar | GenBank | C1 |
| AY342690 | Mar | GenBank | C1 |
| AY342747 | Oct | GenBank | C1 |
| AY342748 | May | GenBank | C1 |
| AY342749 | Jun | GenBank | C1 |
| AY342750 | Sep | GenBank | C1 |
| AY342751 | May | GenBank | C1 |
| AY342752 | Jun | GenBank | C1 |
| AY342753 | May | GenBank | C1 |
| AY342754 | Dec | GenBank | C1 |
| AY342755 | Mar | GenBank | C1 |
| AY342756 | Jun | GenBank | C1 |
| AY342757 | Jul | GenBank | C1 |
| AY342758 | Jul | GenBank | C1 |
| AY342759 | Jun | GenBank | C1 |
| AY342760 | Jun | GenBank | C1 |
| AY342761 | Jun | GenBank | C1 |
| AY342762 | Sep | GenBank | C1 |
| AY342763 | Sep | GenBank | C1 |
| AY342764 | May | GenBank | C1 |
| AY342765 | Jun | GenBank | C1 |
| AY342766 | May | GenBank | C1 |
| AY342767 | Apr | GenBank | C1 |
| AY342768 | Feb | GenBank | C1 |
| AY342769 | Feb | GenBank | C1 |
| AY342770 | Feb | GenBank | C1 |
| AY342771 | Mar | GenBank | C1 |
| AY342772 | Mar | GenBank | C1 |
| AY342773 | Mar | GenBank | C1 |
| AY342774 | Apr | GenBank | C1 |
| AY342775 | Dec | GenBank | C1 |
| AY342776 | Jun | GenBank | C1 |
| AY342777 | May | GenBank | C1 |
| AY342778 | Nov | GenBank | C1 |
| AY342780 | May | GenBank | C1 |
| AY342782 | Dec | GenBank | C1 |
| AY342783 | Mar | GenBank | C1 |
| AY342790 | Jun | GenBank | C1 |
| AY342797 | Sep | GenBank | C1 |
| AY342799 | Oct | GenBank | C1 |
| AY342800 | Nov | GenBank | C1 |
| AY342805 | May | GenBank | C1 |
| AY342806 | Jun | GenBank | C1 |
| AY342810 | Nov | GenBank | C1 |
| AY342815 | Dec | GenBank | C1 |
| AY342830 | Jul | GenBank | C1 |
| AY342831 | May | GenBank | C1 |
| AY342832 | Sep | GenBank | C1 |
| AY342833 | Jul | GenBank | C1 |
| DQ317195 | Aug | GenBank | C2 |
| DQ317196 | Jul | GenBank | C2 |
| DQ317197 | Jun | GenBank | C2 |
| DQ317198 | Mar | GenBank | C2 |
| DQ317199 | Sep | GenBank | C2 |
| DQ317200 | May | GenBank | C2 |
| DQ317201 | Oct | GenBank | C2 |
| DQ317202 | Oct | GenBank | C2 |
| DQ317203 | Jun | GenBank | C1 |
| DQ317204 | Nov | GenBank | C2 |
| EU372170 | Feb | GenBank | C2 |
| EU372171 | Oct | GenBank | C2 |
| EU372172 | Nov | GenBank | C2 |
| EU372173 | Dec | GenBank | C2 |
| EU372174 | Nov | GenBank | C2 |
| FJ868306 | Jul | GenBank | C2 |
| FJ868307 | Nov | GenBank | C2 |
| FJ868347 | Apr | GenBank | C2 |
| FJ868348 | Jan | GenBank | C2 |
| FJ868349 | Jan | GenBank | C2 |
| FJ868350 | Mar | GenBank | C2 |
| FJ868351 | Aug | GenBank | C2 |
| FJ868352 | Aug | GenBank | C2 |
| FJ868353 | Aug | GenBank | C2 |
| FJ868354 | Sep | GenBank | C2 |
| GQ205563 | May | GenBank | C2 |
| GQ205564 | Jul | GenBank | C2 |
| GQ205565 | Jun | GenBank | C2 |
| GQ205566 | Jun | GenBank | C2 |
| GQ205567 | Jul | GenBank | C2 |
| GQ205568 | Jun | GenBank | C2 |
| GQ205569 | Jul | GenBank | C2 |
| GQ205570 | Jun | GenBank | C2 |
| GQ205571 | May | GenBank | C2 |
| GQ205572 | Jun | GenBank | C2 |
| GQ205573 | Jun | GenBank | C2 |
| GQ205574 | Aug | GenBank | C2 |
| GQ205575 | May | GenBank | C2 |
| GQ205576 | Jul | GenBank | C2 |
| GQ205577 | Jun | GenBank | C2 |
| GQ205578 | Jul | GenBank | C2 |
| GQ205579 | Jul | GenBank | C2 |
| GU142897 | Sep | GenBank | C2 |
| GU142898 | Dec | GenBank | C1 |
| GU142899 | Oct | GenBank | C1 |
| GU142900 | Oct | GenBank | C1 |
| GU142901 | Sep | GenBank | C1 |
| GU142902 | Feb | GenBank | C2 |
| HM777023 | Apr | GenBank | C2 |
| JX139826 | Dec | GenBank | C1 |
| JX473479 | Sep | GenBank | A |
| KJ649261 | Dec | GenBank | C2 |
| KJ746497 | Apr | GenBank | C2 |
| KM820892 | Jun | GenBank | C1 |
| KT364218 | Jun | GenBank | C1 |
| KT693321 | Aug | GenBank | C1 |
| KT693322 | Aug | GenBank | C1 |
| KT693323 | Aug | GenBank | C1 |
| KT693324 | Aug | GenBank | C1 |
| KT693325 | Aug | GenBank | C1 |
| KT693326 | Aug | GenBank | C1 |
| KT693327 | Aug | GenBank | C1 |
| KT693328 | Aug | GenBank | C1 |
| KT693329 | Aug | GenBank | C1 |
| KT693330 | Aug | GenBank | C1 |
| KT693331 | Aug | GenBank | C1 |
| KT693332 | Aug | GenBank | C1 |
| KT853019 | Jun | GenBank | C1 |
| KU216191 | Jun | GenBank | C2 |
| KU216192 | Jun | GenBank | C2 |
| KU216193 | Jun | GenBank | C2 |
| KU216194 | Jun | GenBank | C2 |
| KU216195 | Jun | GenBank | C2 |
| KU216196 | Jun | GenBank | C2 |
| KU216197 | Jun | GenBank | C2 |
| KU216198 | Jun | GenBank | C2 |
| KU216199 | Jun | GenBank | C2 |
| KU216200 | Jun | GenBank | C2 |
| KU216201 | Jun | GenBank | C2 |
| KU216202 | Jun | GenBank | C2 |
| KU216203 | Jun | GenBank | C2 |
| KU216204 | Jun | GenBank | C2 |
| KU216205 | Jun | GenBank | C2 |
| KU297237 | Jul | GenBank | C1 |
| KU555717 | Jul | GenBank | C1 |
| KU574621 | Jun | GenBank | C2 |
| KX139439 | Oct | GenBank | C1 |
| KX139441 | Oct | GenBank | C1 |
| KX139444 | Nov | GenBank | C1 |
| KX139446 | Nov | GenBank | C1 |
| KX139447 | Nov | GenBank | C1 |
| KX139448 | Nov | GenBank | C1 |
| KX139449 | Nov | GenBank | C1 |
| KX139450 | Nov | GenBank | C1 |
| KX139451 | Nov | GenBank | C1 |
| KX139452 | Nov | GenBank | C1 |
| KX139453 | Nov | GenBank | C1 |
| KX139454 | Nov | GenBank | C1 |
| KX139455 | Nov | GenBank | C1 |
| KX139456 | Nov | GenBank | C1 |
| KX139457 | Nov | GenBank | C1 |
| KX139458 | Nov | GenBank | C1 |
| KX767786 | Jul | GenBank | C2 |
| KY303773 | Jul | GenBank | C2 |
| KY303774 | Jul | GenBank | C2 |
| KY303775 | Apr | GenBank | C2 |
| KY303776 | Jul | GenBank | C2 |
| KY303777 | Jul | GenBank | C2 |
| KY303778 | Aug | GenBank | C2 |
| KY303779 | Aug | GenBank | C2 |
| KY303780 | Apr | GenBank | C2 |
| KY303781 | Aug | GenBank | C2 |
| KY303782 | May | GenBank | C2 |
| KY303783 | Jul | GenBank | C2 |
| KY303784 | Aug | GenBank | C2 |
| KY303785 | Jul | GenBank | C2 |
| KY303786 | Aug | GenBank | C2 |
| KY303787 | Sep | GenBank | C2 |
| KY303788 | Jul | GenBank | C2 |
| KY303789 | Aug | GenBank | C2 |
| KY303790 | Jul | GenBank | C2 |
| KY303791 | Jul | GenBank | C2 |
| KY303792 | Aug | GenBank | C2 |
| KY303793 | Jul | GenBank | C2 |
| KY303794 | Jul | GenBank | C2 |
| KY303795 | Jul | GenBank | C2 |
| KY303796 | Jul | GenBank | C2 |
| KY303797 | Aug | GenBank | C2 |
| KY303798 | Jul | GenBank | C2 |
| KY303799 | Jul | GenBank | C2 |
| KY303800 | Aug | GenBank | C2 |
| KY303801 | Sep | GenBank | C2 |
| KY303802 | Aug | GenBank | C2 |
| KY303803 | Jul | GenBank | C2 |
| KY303804 | May | GenBank | C2 |
| KY303805 | May | GenBank | C2 |
| KY303806 | Aug | GenBank | C2 |
| KY303807 | May | GenBank | C2 |
| KY303808 | Aug | GenBank | C2 |
| KY303809 | Aug | GenBank | C2 |
| KY303810 | Jul | GenBank | C2 |
| KY303811 | Aug | GenBank | C2 |
| KY303812 | Aug | GenBank | C2 |
| KY303813 | Jul | GenBank | C2 |
| KY303814 | Aug | GenBank | C2 |
| KY303815 | May | GenBank | C2 |
| KY303816 | Jul | GenBank | C2 |
| KY303817 | Jul | GenBank | C2 |
| KY303818 | Jul | GenBank | C2 |
| KY303819 | Jul | GenBank | C2 |
| KY303820 | Aug | GenBank | C2 |
| KY303821 | Aug | GenBank | C2 |
| KY303822 | Sep | GenBank | C2 |
| KY303823 | Jul | GenBank | C2 |
| KY303824 | Jul | GenBank | C2 |
| KY303825 | Jul | GenBank | C2 |
| KY303826 | Jul | GenBank | C2 |
| KY303827 | Jul | GenBank | C2 |
| KY303828 | May | GenBank | C2 |
| KY303829 | Aug | GenBank | C2 |
| KY828851 | Jul | GenBank | C2 |
| KY828852 | Jul | GenBank | C2 |
| LC106305 | Oct | GenBank | C2 |
| LC106306 | Oct | GenBank | C2 |
| LC383283 | Nov | GenBank | C2 |
| LN713457 | Dec | GenBank | C2 |
| MF160175 | Oct | GenBank | C2 |
| MF160210 | Sep | GenBank | C2 |
| MF160229 | Oct | GenBank | C2 |
| MF160232 | Oct | GenBank | C2 |
| MF160242 | Oct | GenBank | C2 |
| MF467308 | Oct | GenBank | C2 |
| MF467309 | Oct | GenBank | C2 |
| MF467310 | Oct | GenBank | C2 |
| MF467311 | Oct | GenBank | C2 |
| MF467312 | Nov | GenBank | C2 |
| MF467313 | Nov | GenBank | C2 |
| MF467314 | Nov | GenBank | C2 |
| MF589254 | Aug | GenBank | C2 |
| MF589290 | Dec | GenBank | C2 |
| MF589298 | Apr | GenBank | C2 |
| MF990301 | Apr | GenBank | B |
| MG720242 | Jun | GenBank | C2 |
| MG720243 | Jun | GenBank | C2 |
| MG720244 | Jun | GenBank | C2 |
| MG720245 | Jun | GenBank | C2 |
| MG720246 | Jul | GenBank | C2 |
| MG720247 | Aug | GenBank | C2 |
| MG720248 | Aug | GenBank | C2 |
| MG720249 | Aug | GenBank | C2 |
| MG720250 | Aug | GenBank | C2 |
| MG720251 | Nov | GenBank | C2 |
| MG720252 | May | GenBank | C2 |
| MG720253 | Apr | GenBank | C2 |
| MG720254 | May | GenBank | C2 |
| MG720255 | May | GenBank | C2 |
| MG720256 | May | GenBank | C2 |
| MG720257 | Jul | GenBank | C2 |
| MG720258 | Aug | GenBank | C2 |
| MG720259 | Aug | GenBank | C2 |
| MG720260 | Jul | GenBank | C2 |
| MG720261 | Jul | GenBank | C2 |
| MG773498 | Feb | GenBank | C2 |
| MG773499 | May | GenBank | C2 |
| MG773500 | May | GenBank | C2 |
| MG773501 | May | GenBank | C2 |
| MG773502 | Jul | GenBank | C2 |
| MG773503 | Aug | GenBank | C2 |
| MG773504 | Jul | GenBank | C2 |
| MG773505 | Aug | GenBank | C2 |
| MG773506 | Aug | GenBank | C2 |
| MG773507 | Aug | GenBank | C2 |
| MG773508 | Apr | GenBank | C2 |
| MG773509 | Sep | GenBank | C2 |
| MG773510 | Aug | GenBank | C2 |
| MH118976 | Nov | GenBank | C2 |
| MH118977 | Nov | GenBank | C2 |
| MH118978 | Nov | GenBank | C2 |
| MH118979 | Nov | GenBank | C2 |
| MH716181 | May | GenBank | C2 |
| MK256761 | Jul | GenBank | C2 |
| MK256762 | Jul | GenBank | C2 |
| MK531854 | May | GenBank | B |
| MK531855 | May | GenBank | B |
| MK836155 | Aug | GenBank | C2 |
| MK836172 | Oct | GenBank | C2 |
| MN052953 | Aug | GenBank | C2 |
| MN052954 | Jun | GenBank | C2 |
| MN166092 | Jul | GenBank | C2 |
| MN215884 | May | GenBank | C2 |
| MN337405 | Jun | GenBank | C2 |
| MN688218 | Apr | GenBank | C2 |
| MN737181 | Jul | GenBank | C2 |
| MN737182 | Jul | GenBank | C2 |
| MN737183 | Jul | GenBank | C2 |
| MN737184 | Jul | GenBank | C2 |
| MN737185 | Jul | GenBank | C2 |
| MN737186 | Jul | GenBank | C2 |
| MN737187 | Jul | GenBank | C2 |
| MN737188 | Jul | GenBank | C2 |
| MN737189 | Jul | GenBank | C2 |
| MN737190 | Jul | GenBank | C2 |
| MN832717 | Jul | GenBank | C2 |
| MN832718 | Jul | GenBank | C2 |
| MT614259 | Nov | GenBank | C2 |
| MT641376 | Oct | GenBank | C2 |
| MT641383 | Aug | GenBank | C2 |
| MT641413 | Jul | GenBank | C2 |
| MT641418 | Jul | GenBank | C2 |
| MT755385 | Jun | GenBank | C2 |
| MW731975 | Feb | GenBank | C2 |
| MW731976 | Feb | GenBank | C2 |
| MW731977 | Aug | GenBank | C2 |
| MW731978 | Sep | GenBank | C2 |
| MW731979 | Sep | GenBank | C2 |
| MW731980 | Feb | GenBank | C2 |
| MW731981 | Jun | GenBank | C2 |
| OK585080 | Apr | GenBank | C2 |
| OQ791557 | Mar | GenBank | C2 |
| OQ791558 | Mar | GenBank | C2 |
| OQ791559 | Mar | GenBank | C2 |
| OQ791562 | May | GenBank | C2 |
| OQ791564 | Aug | GenBank | C2 |
| OQ842421 | Aug | GenBank | C1 |
| OQ842422 | Aug | GenBank | C1 |

Jan: January; Feb: February; Mar: March; Apr: April; May: May; Jun: June; Jul: July; Aug:August; Sep:September; Oct: October; Nov: November; Dec: December.
