# Supplementary material for: Circulated echovirus 18 strains in Guangdong Province and worldwide: A novel perspective on genetic diversity and recombination patterns
Source: Virulence. 2025 Jul 15;16(1):2534519. doi: 10.1080/21505594.2025.2534519 (PMC12296116; doi:10.1080/21505594.2025.2534519)
Supplement: Supplemental Material [file KVIR_A_2534519_SM5328.zip › Supplementary File_1_Table_S5.docx]

**Supplementary Table S5.** Analysis of 256 sequences to infer isolated sources between 2000 and 2022.

| Year | Stool | CSF | Respiratory sample | Rectal swab | Blood sample | Sewage | Total |
| --- | --- | --- | --- | --- | --- | --- | --- |
| 2000 | 4 | 0 | 1 | 0 | 0 | 0 | 5 |
| 2001 | 0 | 0 | 0 | 0 | 0 | 0 | 0 |
| 2002 | 5 | 0 | 0 | 0 | 0 | 0 | 5 |
| 2003 | 5 | 0 | 0 | 0 | 0 | 0 | 5 |
| 2004 | 0 | 0 | 7 | 0 | 0 | 0 | 7 |
| 2005 | 7 | 9 | 0 | 0 | 0 | 0 | 16 |
| 2006 | 1 | 11 | 2 | 0 | 0 | 0 | 14 |
| 2007 | 2 | 0 | 0 | 0 | 0 | 0 | 2 |
| 2008 | 3 | 0 | 0 | 0 | 0 | 0 | 3 |
| 2009 | 3 | 0 | 0 | 0 | 0 | 0 | 3 |
| 2010 | 16 | 4 | 0 | 0 | 0 | 0 | 20 |
| 2011 | 2 | 5 | 0 | 0 | 0 | 1 | 8 |
| 2012 | 0 | 5 | 0 | 0 | 2 | 0 | 7 |
| 2013 | 0 | 2 | 1 | 0 | 0 | 0 | 3 |
| 2014 | 6 | 0 | 9 | 0 | 0 | 3 | 18 |
| 2015 | 2 | 7 | 3 | 4 | 4 | 1 | 21 |
| 2016 | 4 | 0 | 2 | 0 | 0 | 0 | 6 |
| 2017 | 2 | 15 | 0 | 0 | 0 | 0 | 17 |
| 2018 | 0 | 14 | 3 | 0 | 1 | 2 | 20 |
| 2019 | 15 | 46 | 7 | 0 | 0 | 0 | 68 |
| 2020 | 2 | 0 | 0 | 0 | 0 | 0 | 2 |
| 2021 | 3 | 0 | 0 | 0 | 0 | 0 | 3 |
| 2022 | 3 | 0 | 0 | 0 | 0 | 0 | 3 |
