# Supplementary material for: Circulated echovirus 18 strains in Guangdong Province and worldwide: A novel perspective on genetic diversity and recombination patterns
Source: Virulence. 2025 Jul 15;16(1):2534519. doi: 10.1080/21505594.2025.2534519 (PMC12296116; doi:10.1080/21505594.2025.2534519)
Supplement: Supplemental Material [file KVIR_A_2534519_SM5328.zip › Supplementary File_1_Table_S8.docx]

**Supplementary Table S8.** Information on 79 E18 sequences and closely related E30 sequences used for recombination analysis.

| Accession | Year | Country | Source | Genotype |
| --- | --- | --- | --- | --- |
| OQ842422 | 2012 | USA | GenBank | E18 |
| OQ842421 | 2012 | USA | GenBank | E18 |
| OQ791564 | 2015 | USA | GenBank | E18 |
| OQ791562 | 2015 | USA | GenBank | E18 |
| OQ791559 | 2015 | USA | GenBank | E18 |
| OQ791558 | 2015 | USA | GenBank | E18 |
| OQ791557 | 2015 | USA | GenBank | E18 |
| MW481634 | 2019 | China | GenBank | E18 |
| MT755385 | 2019 | China | GenBank | E18 |
| MT350224 | 2019 | China | GenBank | E18 |
| MN832718 | 2019 | China | GenBank | E18 |
| MN832717 | 2019 | China | GenBank | E18 |
| MN815813 | 2018 | China | GenBank | E18 |
| MN815812 | 2019 | China | GenBank | E18 |
| MN815811 | 2018 | China | GenBank | E18 |
| MN815810 | 2018 | China | GenBank | E18 |
| MN808794 | 2019 | China | GenBank | E18 |
| MN808793 | 2019 | China | GenBank | E18 |
| MN808792 | 2019 | China | GenBank | E18 |
| MN792654 | 2019 | China | GenBank | E18 |
| MN749146 | 2015 | USA | GenBank | E18 |
| MN749143 | 2015 | USA | GenBank | E18 |
| MN737190 | 2019 | China | GenBank | E18 |
| MN737189 | 2019 | China | GenBank | E18 |
| MN737188 | 2019 | China | GenBank | E18 |
| MN737187 | 2019 | China | GenBank | E18 |
| MN737186 | 2019 | China | GenBank | E18 |
| MN737185 | 2019 | China | GenBank | E18 |
| MN737184 | 2019 | China | GenBank | E18 |
| MN737183 | 2019 | China | GenBank | E18 |
| MN737182 | 2019 | China | GenBank | E18 |
| MN737181 | 2019 | China | GenBank | E18 |
| MN337405 | 2019 | China | GenBank | E18 |
| MN215884 | 2019 | China | GenBank | E18 |
| MN166092 | 2015 | USA | GenBank | E18 |
| MG720261 | 2015 | China | GenBank | E18 |
| MG720260 | 2015 | China | GenBank | E18 |
| MG720259 | 2015 | China | GenBank | E18 |
| MG720258 | 2015 | China | GenBank | E18 |
| MG720257 | 2015 | China | GenBank | E18 |
| MG720256 | 2015 | China | GenBank | E18 |
| MF990301 | 2016 | Ethiopia | GenBank | E18 |
| MF838733 | 2011 | Australia | GenBank | E18 |
| MF678301 | 2008 | Australia | GenBank | E18 |
| KY828852 | 2016 | China | GenBank | E18 |
| KY828851 | 2016 | China | GenBank | E18 |
| KX767786 | 2015 | China | GenBank | E18 |
| KX139458 | 2010 | Germany | GenBank | E18 |
| KX139457 | 2010 | Germany | GenBank | E18 |
| KX139456 | 2010 | Germany | GenBank | E18 |
| KX139455 | 2010 | Germany | GenBank | E18 |
| KX139454 | 2010 | Germany | GenBank | E18 |
| KX139453 | 2010 | Germany | GenBank | E18 |
| KX139452 | 2010 | Germany | GenBank | E18 |
| KX139451 | 2010 | Germany | GenBank | E18 |
| KX139450 | 2010 | Germany | GenBank | E18 |
| KX139449 | 2010 | Germany | GenBank | E18 |
| KX139448 | 2010 | Germany | GenBank | E18 |
| KX139447 | 2010 | Germany | GenBank | E18 |
| KX139446 | 2010 | Germany | GenBank | E18 |
| KU574621 | 2010 | Thailand | GenBank | E18 |
| HM777023 | 2005 | South Korea | GenBank | E18 |
| AF317694 | 2000 | Sweden | GenBank | E18 |
| PP891443 | 2022 | China | This study | E18 |
| PP891442 | 2022 | China | This study | E18 |
| PP891441 | 2022 | China | This study | E18 |
| PP891440 | 2019 | China | This study | E18 |
| PP891439 | 2019 | China | This study | E18 |
| PP891438 | 2019 | China | This study | E18 |
| PP891437 | 2019 | China | This study | E18 |
| MN153801 | 2017 | USA | GenBank | E30 |
| MK238483 | 2017 | USA | GenBank | E30 |
| MZ229659 | 2019 | China | GenBank | E30 |
| MZ229660 | 2019 | China | GenBank | E30 |
| OQ842429 | 2017 | USA | GenBank | E30 |
| MW586892 | 2017 | New Zealand | GenBank | E30 |
| OM677620 | 2021 | China | GenBank | E30 |
| MW080377 | 2016 | China | GenBank | E30 |
| MW080372 | 2016 | China | GenBank | E30 |

USA: United States of America.
