# Supplementary material for: Circulated echovirus 18 strains in Guangdong Province and worldwide: A novel perspective on genetic diversity and recombination patterns
Source: Virulence. 2025 Jul 15;16(1):2534519. doi: 10.1080/21505594.2025.2534519 (PMC12296116; doi:10.1080/21505594.2025.2534519)
Supplement: Supplemental Material [file KVIR_A_2534519_SM5328.zip › Supplementary File_1_Table_S9.docx]

**Supplementary Table S9.** Information of 77 sequences for construction of heatmap and detection of recombination.

| Accession | Country | Year | Source | Genotype |
| --- | --- | --- | --- | --- |
| OQ842422 | USA | 2012 | GenBank | E18 |
| OQ842421 | USA | 2012 | GenBank | E18 |
| OQ791564 | USA | 2015 | GenBank | E18 |
| OQ791562 | USA | 2015 | GenBank | E18 |
| OQ791559 | USA | 2015 | GenBank | E18 |
| OQ791558 | USA | 2015 | GenBank | E18 |
| OQ791557 | USA | 2015 | GenBank | E18 |
| MW481634 | China | 2019 | GenBank | E18 |
| MT755385 | China | 2019 | GenBank | E18 |
| MT350224 | China | 2019 | GenBank | E18 |
| MN832718 | China | 2019 | GenBank | E18 |
| MN832717 | China | 2019 | GenBank | E18 |
| MN815813 | China | 2018 | GenBank | E18 |
| MN815812 | China | 2019 | GenBank | E18 |
| MN815811 | China | 2018 | GenBank | E18 |
| MN815810 | China | 2018 | GenBank | E18 |
| MN808794 | China | 2019 | GenBank | E18 |
| MN808793 | China | 2019 | GenBank | E18 |
| MN808792 | China | 2019 | GenBank | E18 |
| MN792654 | China | 2019 | GenBank | E18 |
| MN749146 | USA | 2015 | GenBank | E18 |
| MN749143 | USA | 2015 | GenBank | E18 |
| MN737190 | China | 2019 | GenBank | E18 |
| MN737189 | China | 2019 | GenBank | E18 |
| MN737188 | China | 2019 | GenBank | E18 |
| MN737187 | China | 2019 | GenBank | E18 |
| MN737186 | China | 2019 | GenBank | E18 |
| MN737185 | China | 2019 | GenBank | E18 |
| MN737184 | China | 2019 | GenBank | E18 |
| MN737183 | China | 2019 | GenBank | E18 |
| MN737182 | China | 2019 | GenBank | E18 |
| MN737181 | China | 2019 | GenBank | E18 |
| MN337405 | China | 2019 | GenBank | E18 |
| MN215884 | China | 2019 | GenBank | E18 |
| MN166092 | USA | 2015 | GenBank | E18 |
| MG720261 | China | 2015 | GenBank | E18 |
| MG720260 | China | 2015 | GenBank | E18 |
| MG720259 | China | 2015 | GenBank | E18 |
| MG720258 | China | 2015 | GenBank | E18 |
| MG720257 | China | 2015 | GenBank | E18 |
| MG720256 | China | 2015 | GenBank | E18 |
| MF990301 | Ethiopia | 2016 | GenBank | E18 |
| MF838733 | Australia | 2011 | GenBank | E18 |
| KY828852 | China | 2016 | GenBank | E18 |
| KY828851 | China | 2016 | GenBank | E18 |
| KX767786 | China | 2015 | GenBank | E18 |
| KX139458 | Germany | 2010 | GenBank | E18 |
| KX139457 | Germany | 2010 | GenBank | E18 |
| KX139456 | Germany | 2010 | GenBank | E18 |
| KX139455 | Germany | 2010 | GenBank | E18 |
| KX139454 | Germany | 2010 | GenBank | E18 |
| KX139453 | Germany | 2010 | GenBank | E18 |
| KX139452 | Germany | 2010 | GenBank | E18 |
| KX139451 | Germany | 2010 | GenBank | E18 |
| KX139450 | Germany | 2010 | GenBank | E18 |
| KX139449 | Germany | 2010 | GenBank | E18 |
| KX139448 | Germany | 2010 | GenBank | E18 |
| KX139447 | Germany | 2010 | GenBank | E18 |
| KX139446 | Germany | 2010 | GenBank | E18 |
| HM777023 | South Korea | 2005 | GenBank | E18 |
| AF317694 | Sweden | 2000 | GenBank | E18 |
| PP891443 | China | 2022 | This study | E18 |
| PP891442 | China | 2022 | This study | E18 |
| PP891441 | China | 2022 | This study | E18 |
| PP891440 | China | 2019 | This study | E18 |
| PP891439 | China | 2019 | This study | E18 |
| PP891438 | China | 2019 | This study | E18 |
| PP891437 | China | 2019 | This study | E18 |
| MN153801 | USA | 2017 | GenBank | E30 |
| MK238483 | USA | 2017 | GenBank | E30 |
| MZ229659 | China | 2019 | GenBank | E30 |
| MZ229660 | China | 2019 | GenBank | E30 |
| OQ842429 | USA | 2017 | GenBank | E30 |
| MW586892 | New Zealand | 2017 | GenBank | E30 |
| OM677620 | China | 2021 | GenBank | E30 |
| MW080377 | China | 2016 | GenBank | E30 |
| MW080372 | China | 2016 | GenBank | E30 |

USA: United States of America.
