# Supplementary material for: Circulated echovirus 18 strains in Guangdong Province and worldwide: A novel perspective on genetic diversity and recombination patterns
Source: Virulence. 2025 Jul 15;16(1):2534519. doi: 10.1080/21505594.2025.2534519 (PMC12296116; doi:10.1080/21505594.2025.2534519)
Supplement: Supplemental Material [file KVIR_A_2534519_SM5328.zip › Supplementary File_2.docx]

**Supplementary File 2**

**Supplemental Figure S1.** The geographic distribution of 229 E18 sequences in China from 2000 to 2022. The numbers in brackets represent the E18 sequences in each province of China.


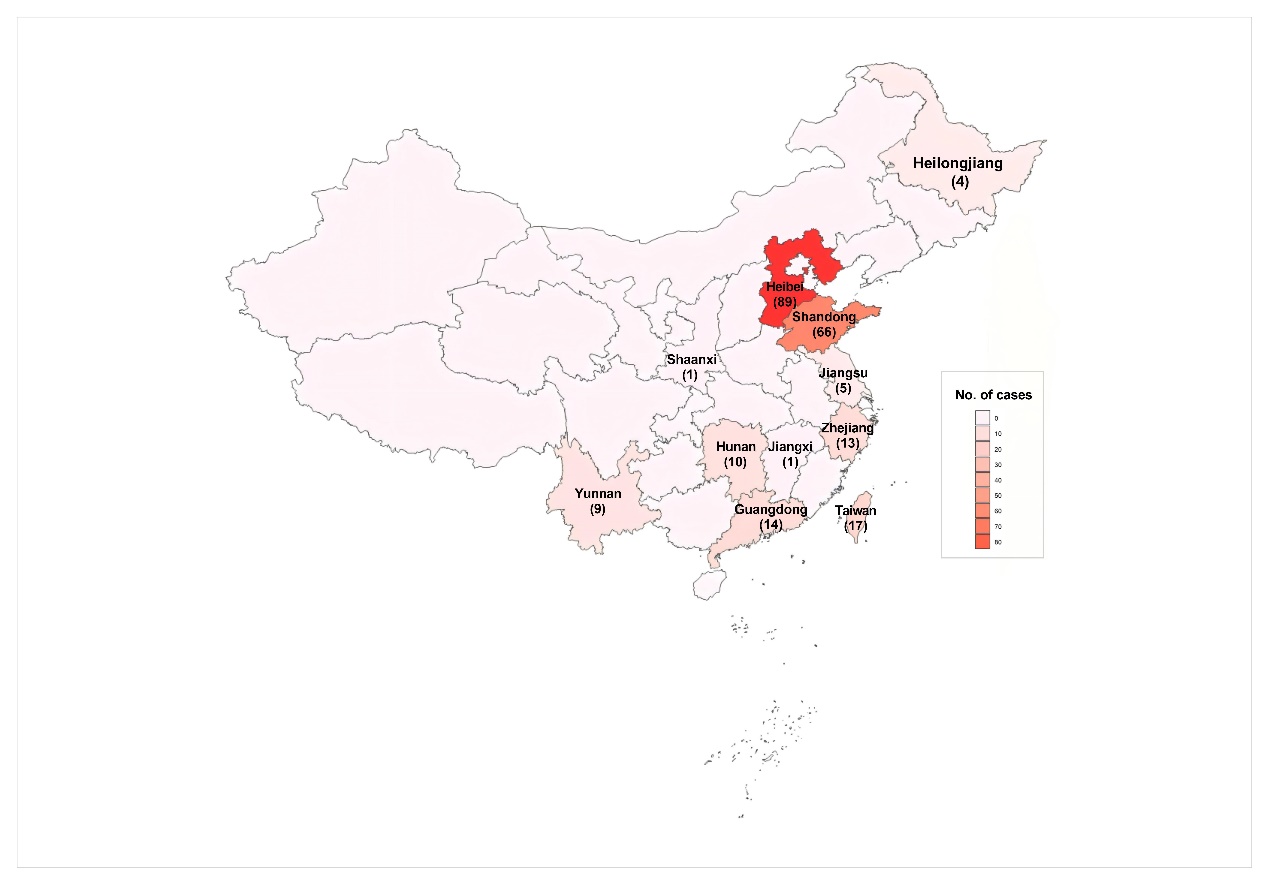


**Supplemental Figure S2.** Neighbor-joining phylogenetic trees were constructed separately for the P1 (a), P2 (b), and P3 (c) regions of the genomes of E18, as well as for closely related E30 strains. The branches of the trees are color-coded based on their country of isolation. The E18 sequences newly generated in this study are highlighted in bold red (n=7), while an additional 63 E18 sequences retrieved from the GenBank database are also included in the analysis. The E30 genomes are indicated in bold black (n=9).

**
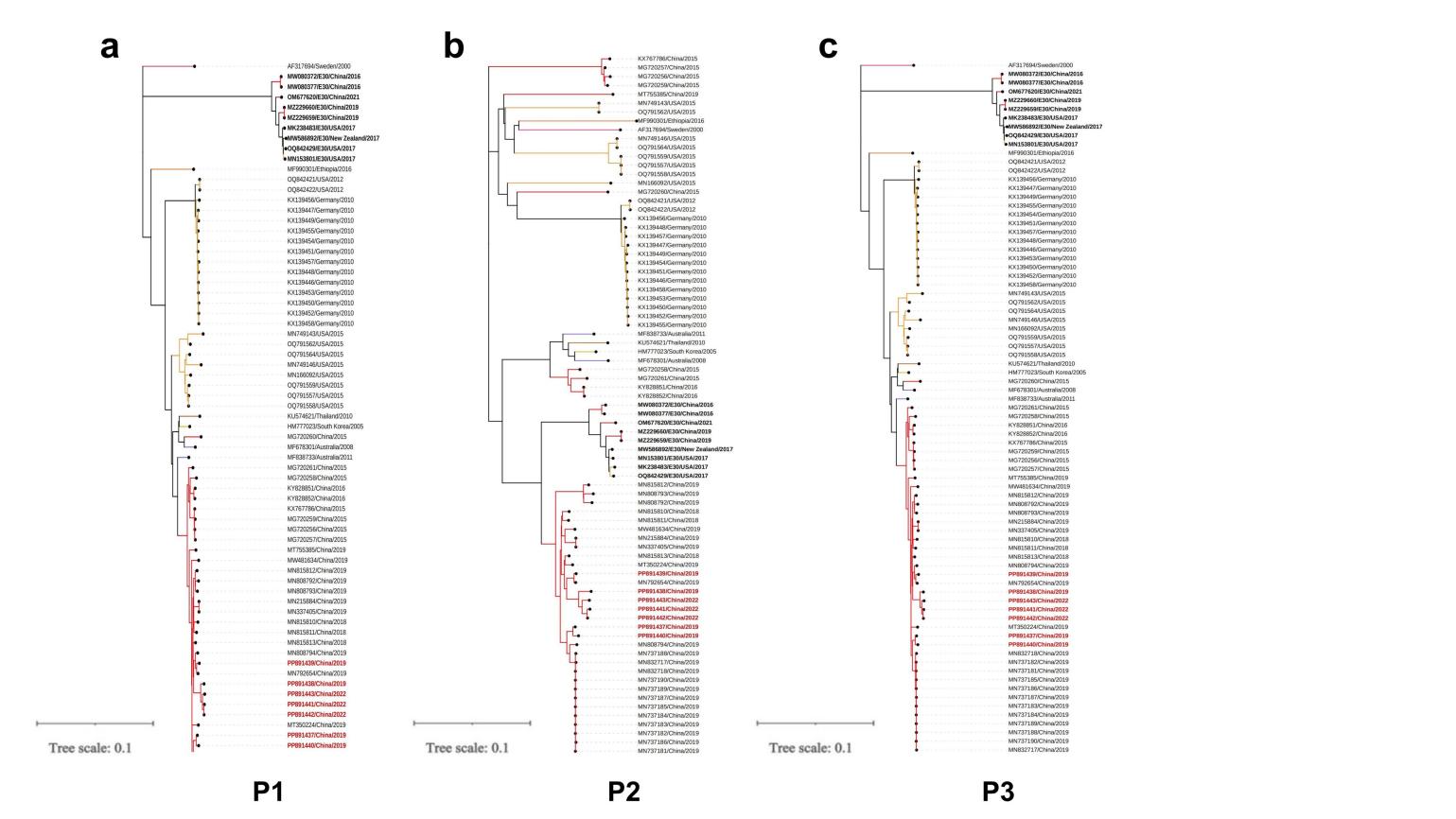
**
