# Supplementary material for: Circulated echovirus 18 strains in Guangdong Province and worldwide: A novel perspective on genetic diversity and recombination patterns
Source: Virulence. 2025 Jul 15;16(1):2534519. doi: 10.1080/21505594.2025.2534519 (PMC12296116; doi:10.1080/21505594.2025.2534519)
Supplement: Supplemental Material [file KVIR_A_2534519_SM5328.zip › Supplementary Flie_1_Table_S6.docx]

**Supplementary Table S6.** Information of 290 complete VP1 gene sequences of E18 for phylogeny relationship inference

| Accession | Year | Country | Source | Genotype |
| --- | --- | --- | --- | --- |
| OQ842422 | 2012 | USA | GenBank | C1 |
| OQ842421 | 2012 | USA | GenBank | C1 |
| OQ791564 | 2015 | USA | GenBank | C2 |
| OQ791562 | 2015 | USA | GenBank | C2 |
| OQ791559 | 2015 | USA | GenBank | C2 |
| OQ791558 | 2015 | USA | GenBank | C2 |
| OQ791557 | 2015 | USA | GenBank | C2 |
| MW481634 | 2019 | China | GenBank | C2 |
| MT950636 | 2018 | China | GenBank | C2 |
| MT950635 | 2018 | China | GenBank | C2 |
| MT950634 | 2018 | China | GenBank | C2 |
| MT950633 | 2018 | China | GenBank | C2 |
| MT950632 | 2018 | China | GenBank | C2 |
| MT950631 | 2018 | China | GenBank | C2 |
| MT950630 | 2018 | China | GenBank | C2 |
| MT950629 | 2018 | China | GenBank | C2 |
| MT950628 | 2018 | China | GenBank | C2 |
| MT950627 | 2018 | China | GenBank | C2 |
| MT950626 | 2018 | China | GenBank | C2 |
| MT950598 | 2019 | China | GenBank | C2 |
| MT950597 | 2019 | China | GenBank | C2 |
| MT950596 | 2019 | China | GenBank | C2 |
| MT950595 | 2019 | China | GenBank | C2 |
| MT950594 | 2019 | China | GenBank | C2 |
| MT950593 | 2019 | China | GenBank | C2 |
| MT950592 | 2019 | China | GenBank | C2 |
| MT950590 | 2019 | China | GenBank | C2 |
| MT950588 | 2019 | China | GenBank | C2 |
| MT950587 | 2019 | China | GenBank | C2 |
| MT950586 | 2019 | China | GenBank | C2 |
| MT950585 | 2019 | China | GenBank | C2 |
| MT950584 | 2019 | China | GenBank | C2 |
| MT950583 | 2019 | China | GenBank | C2 |
| MT950582 | 2019 | China | GenBank | C2 |
| MT950581 | 2019 | China | GenBank | C2 |
| MT950580 | 2019 | China | GenBank | C2 |
| MT950578 | 2019 | China | GenBank | C2 |
| MT950577 | 2019 | China | GenBank | C2 |
| MT950576 | 2019 | China | GenBank | C2 |
| MT950574 | 2019 | China | GenBank | C2 |
| MT950573 | 2019 | China | GenBank | C2 |
| MT950572 | 2019 | China | GenBank | C2 |
| MT950571 | 2019 | China | GenBank | C2 |
| MT950570 | 2019 | China | GenBank | C2 |
| MT950568 | 2019 | China | GenBank | C2 |
| MT950566 | 2019 | China | GenBank | C2 |
| MT950563 | 2019 | China | GenBank | C2 |
| MT950562 | 2019 | China | GenBank | C2 |
| MT950559 | 2019 | China | GenBank | C2 |
| MT950558 | 2019 | China | GenBank | C2 |
| MT950556 | 2019 | China | GenBank | C2 |
| MT950553 | 2019 | China | GenBank | C2 |
| MT950552 | 2019 | China | GenBank | C2 |
| MT950550 | 2019 | China | GenBank | C2 |
| MT950549 | 2019 | China | GenBank | C2 |
| MT950546 | 2019 | China | GenBank | C2 |
| MT950544 | 2019 | China | GenBank | C2 |
| MT755385 | 2019 | China | GenBank | C2 |
| MT641418 | 2018 | UK | GenBank | C2 |
| MT641413 | 2018 | UK | GenBank | C2 |
| MT641383 | 2017 | UK | GenBank | C2 |
| MT641376 | 2017 | UK | GenBank | C2 |
| MT350224 | 2019 | China | GenBank | C2 |
| MN896914 | 2019 | USA | GenBank | C2 |
| MN896910 | 2019 | USA | GenBank | C2 |
| MN896909 | 2019 | USA | GenBank | C2 |
| MN896908 | 2019 | USA | GenBank | C2 |
| MN832718 | 2019 | China | GenBank | C2 |
| MN832717 | 2019 | China | GenBank | C2 |
| MN815813 | 2018 | China | GenBank | C2 |
| MN815812 | 2019 | China | GenBank | C2 |
| MN815811 | 2018 | China | GenBank | C2 |
| MN815810 | 2018 | China | GenBank | C2 |
| MN808794 | 2019 | China | GenBank | C2 |
| MN808793 | 2019 | China | GenBank | C2 |
| MN808792 | 2019 | China | GenBank | C2 |
| MN792654 | 2019 | China | GenBank | C2 |
| MN749146 | 2015 | USA | GenBank | C2 |
| MN749143 | 2015 | USA | GenBank | C2 |
| MN737190 | 2019 | China | GenBank | C2 |
| MN737189 | 2019 | China | GenBank | C2 |
| MN737188 | 2019 | China | GenBank | C2 |
| MN737187 | 2019 | China | GenBank | C2 |
| MN737186 | 2019 | China | GenBank | C2 |
| MN737185 | 2019 | China | GenBank | C2 |
| MN737184 | 2019 | China | GenBank | C2 |
| MN737183 | 2019 | China | GenBank | C2 |
| MN737182 | 2019 | China | GenBank | C2 |
| MN737181 | 2019 | China | GenBank | C2 |
| MN688218 | 2019 | China | GenBank | C2 |
| MN541053 | 2018 | China | GenBank | C2 |
| MN541049 | 2018 | China | GenBank | C2 |
| MN337405 | 2019 | China | GenBank | C2 |
| MN215884 | 2019 | China | GenBank | C2 |
| MN166092 | 2015 | USA | GenBank | C2 |
| MK256762 | 2015 | China | GenBank | C2 |
| MK256761 | 2015 | China | GenBank | C2 |
| MH118977 | 2017 | China | GenBank | C2 |
| MG720261 | 2015 | China | GenBank | C2 |
| MG720260 | 2015 | China | GenBank | C2 |
| MG720259 | 2015 | China | GenBank | C2 |
| MG720258 | 2015 | China | GenBank | C2 |
| MG720257 | 2015 | China | GenBank | C2 |
| MG720256 | 2015 | China | GenBank | C2 |
| MG720255 | 2015 | China | GenBank | C2 |
| MG720254 | 2015 | China | GenBank | C2 |
| MG720253 | 2015 | China | GenBank | C2 |
| MG720252 | 2015 | China | GenBank | C2 |
| MG720251 | 2015 | China | GenBank | C2 |
| MG720250 | 2015 | China | GenBank | C2 |
| MG720249 | 2015 | China | GenBank | C2 |
| MG720248 | 2015 | China | GenBank | C2 |
| MG720247 | 2015 | China | GenBank | C2 |
| MG720246 | 2015 | China | GenBank | C2 |
| MG720245 | 2015 | China | GenBank | C2 |
| MG720244 | 2015 | China | GenBank | C2 |
| MG720243 | 2015 | China | GenBank | C2 |
| MG720242 | 2016 | China | GenBank | C2 |
| MF990301 | 2016 | Ethiopia | GenBank | B |
| MF838733 | 2011 | Australia | GenBank | C2 |
| MF678301 | 2008 | Australia | GenBank | C2 |
| LT883145 | 2017 | Tunisia | GenBank | C2 |
| LN713457 | 2012 | Tunisia | GenBank | C2 |
| LC707464 | 2021 | China | GenBank | C2 |
| LC707449 | 2021 | China | GenBank | C2 |
| LC707444 | 2021 | China | GenBank | C2 |
| KY828852 | 2016 | China | GenBank | C2 |
| KY828851 | 2016 | China | GenBank | C2 |
| KY303829 | 2015 | China | GenBank | C2 |
| KY303828 | 2015 | China | GenBank | C2 |
| KY303827 | 2015 | China | GenBank | C2 |
| KY303826 | 2015 | China | GenBank | C2 |
| KY303825 | 2015 | China | GenBank | C2 |
| KY303824 | 2015 | China | GenBank | C2 |
| KY303823 | 2015 | China | GenBank | C2 |
| KY303822 | 2015 | China | GenBank | C2 |
| KY303821 | 2015 | China | GenBank | C2 |
| KY303820 | 2015 | China | GenBank | C2 |
| KY303819 | 2015 | China | GenBank | C2 |
| KY303818 | 2015 | China | GenBank | C2 |
| KY303817 | 2015 | China | GenBank | C2 |
| KY303816 | 2015 | China | GenBank | C2 |
| KY303815 | 2015 | China | GenBank | C2 |
| KY303814 | 2015 | China | GenBank | C2 |
| KY303813 | 2015 | China | GenBank | C2 |
| KY303812 | 2015 | China | GenBank | C2 |
| KY303811 | 2015 | China | GenBank | C2 |
| KY303810 | 2015 | China | GenBank | C2 |
| KY303809 | 2015 | China | GenBank | C2 |
| KY303808 | 2015 | China | GenBank | C2 |
| KY303807 | 2015 | China | GenBank | C2 |
| KY303806 | 2015 | China | GenBank | C2 |
| KY303805 | 2015 | China | GenBank | C2 |
| KY303804 | 2015 | China | GenBank | C2 |
| KY303803 | 2015 | China | GenBank | C2 |
| KY303802 | 2015 | China | GenBank | C2 |
| KY303801 | 2015 | China | GenBank | C2 |
| KY303800 | 2015 | China | GenBank | C2 |
| KY303799 | 2015 | China | GenBank | C2 |
| KY303798 | 2015 | China | GenBank | C2 |
| KY303797 | 2015 | China | GenBank | C2 |
| KY303796 | 2015 | China | GenBank | C2 |
| KY303795 | 2015 | China | GenBank | C2 |
| KY303794 | 2015 | China | GenBank | C2 |
| KY303793 | 2015 | China | GenBank | C2 |
| KY303792 | 2015 | China | GenBank | C2 |
| KY303791 | 2015 | China | GenBank | C2 |
| KY303790 | 2015 | China | GenBank | C2 |
| KY303789 | 2015 | China | GenBank | C2 |
| KY303788 | 2015 | China | GenBank | C2 |
| KY303787 | 2015 | China | GenBank | C2 |
| KY303786 | 2015 | China | GenBank | C2 |
| KY303785 | 2015 | China | GenBank | C2 |
| KY303784 | 2015 | China | GenBank | C2 |
| KY303783 | 2015 | China | GenBank | C2 |
| KY303782 | 2015 | China | GenBank | C2 |
| KY303781 | 2015 | China | GenBank | C2 |
| KY303780 | 2015 | China | GenBank | C2 |
| KY303779 | 2015 | China | GenBank | C2 |
| KY303778 | 2015 | China | GenBank | C2 |
| KY303777 | 2015 | China | GenBank | C2 |
| KY303776 | 2015 | China | GenBank | C2 |
| KY303775 | 2015 | China | GenBank | C2 |
| KY303774 | 2015 | China | GenBank | C2 |
| KY303773 | 2015 | China | GenBank | C2 |
| KX767786 | 2015 | China | GenBank | C2 |
| KX139458 | 2010 | Germany | GenBank | C1 |
| KX139457 | 2010 | Germany | GenBank | C1 |
| KX139456 | 2010 | Germany | GenBank | C1 |
| KX139455 | 2010 | Germany | GenBank | C1 |
| KX139454 | 2010 | Germany | GenBank | C1 |
| KX139453 | 2010 | Germany | GenBank | C1 |
| KX139452 | 2010 | Germany | GenBank | C1 |
| KX139451 | 2010 | Germany | GenBank | C1 |
| KX139450 | 2010 | Germany | GenBank | C1 |
| KX139449 | 2010 | Germany | GenBank | C1 |
| KX139448 | 2010 | Germany | GenBank | C1 |
| KX139447 | 2010 | Germany | GenBank | C1 |
| KX139446 | 2010 | Germany | GenBank | C1 |
| KX139444 | 2010 | Germany | GenBank | C1 |
| KX139441 | 2010 | Germany | GenBank | C1 |
| KX139439 | 2010 | Germany | GenBank | C1 |
| KU574621 | 2010 | Thailand | GenBank | C1 |
| KU561040 | 2011 | Netherlands | GenBank | C2 |
| KU561038 | 2011 | Netherlands | GenBank | C2 |
| KU216205 | 2015 | China | GenBank | C2 |
| KU216204 | 2015 | China | GenBank | C2 |
| KU216203 | 2015 | China | GenBank | C2 |
| KU216202 | 2015 | China | GenBank | C2 |
| KU216201 | 2015 | China | GenBank | C2 |
| KU216200 | 2015 | China | GenBank | C2 |
| KU216199 | 2015 | China | GenBank | C2 |
| KU216198 | 2015 | China | GenBank | C2 |
| KU216197 | 2015 | China | GenBank | C2 |
| KU216196 | 2015 | China | GenBank | C2 |
| KU216195 | 2015 | China | GenBank | C2 |
| KU216194 | 2015 | China | GenBank | C2 |
| KU216193 | 2015 | China | GenBank | C2 |
| KU216192 | 2015 | China | GenBank | C2 |
| KU216191 | 2015 | China | GenBank | C2 |
| KU133628 | 2013 | Russia | GenBank | C1 |
| KU133621 | 2013 | Russia | GenBank | C1 |
| KU133609 | 2012 | Russia | GenBank | C1 |
| KU133587 | 2012 | Russia | GenBank | C1 |
| JN203852 | 2011 | India | GenBank | B |
| JN203851 | 2011 | India | GenBank | B |
| JN203850 | 2011 | India | GenBank | B |
| JN203849 | 2011 | India | GenBank | B |
| HM777023 | 2005 | South Korea | GenBank | C2 |
| HG793727 | 2012 | France | GenBank | C2 |
| HG793726 | 2012 | France | GenBank | C2 |
| HG793725 | 2012 | France | GenBank | C2 |
| HG793724 | 2012 | France | GenBank | C1 |
| HG793723 | 2011 | France | GenBank | C1 |
| HG793722 | 2011 | France | GenBank | C1 |
| HG793721 | 2011 | France | GenBank | C1 |
| HG793720 | 2011 | France | GenBank | C2 |
| HG793719 | 2011 | France | GenBank | C1 |
| HG793718 | 2010 | France | GenBank | C2 |
| HG793717 | 2012 | France | GenBank | C2 |
| HF948108 | 2006 | France | GenBank | C1 |
| HF948107 | 2006 | France | GenBank | C1 |
| HF948106 | 2002 | France | GenBank | C1 |
| HF948105 | 2000 | France | GenBank | C1 |
| HF948104 | 2000 | France | GenBank | C1 |
| GU142902 | 1997 | Australia | GenBank | C2 |
| GU142901 | 2001 | Australia | GenBank | C1 |
| GU142900 | 2001 | Australia | GenBank | C1 |
| GU142899 | 2001 | Australia | GenBank | C1 |
| GU142898 | 2000 | Australia | GenBank | C1 |
| GU142897 | 2005 | Australia | GenBank | C2 |
| GQ329813 | 2005 | China | GenBank | A |
| FJ868354 | 2005 | Australia | GenBank | C2 |
| FJ868353 | 2005 | Australia | GenBank | C2 |
| FJ868352 | 2005 | Australia | GenBank | C2 |
| FJ868351 | 2005 | Australia | GenBank | C2 |
| FJ868350 | 2005 | Australia | GenBank | C2 |
| FJ868349 | 2005 | Australia | GenBank | C2 |
| FJ868348 | 2005 | Australia | GenBank | C2 |
| FJ868347 | 2004 | Australia | GenBank | C2 |
| FJ868307 | 2005 | Australia | GenBank | C2 |
| FJ868306 | 2005 | Australia | GenBank | C2 |
| AY208092 | 2002 | Sweden | GenBank | C1 |
| AY208091 | 2002 | Sweden | GenBank | C1 |
| AM711105 | 2006 | France | GenBank | C1 |
| AM711103 | 2006 | France | GenBank | C1 |
| AM711101 | 2006 | France | GenBank | C1 |
| AM711073 | 2006 | France | GenBank | C1 |
| AM711070 | 2006 | France | GenBank | C1 |
| AM711068 | 2006 | France | GenBank | C1 |
| AM711028 | 2005 | France | GenBank | C2 |
| AM711024 | 2005 | France | GenBank | C2 |
| AM711021 | 2005 | France | GenBank | C2 |
| AM236984 | 2005 | France | GenBank | C2 |
| AM236978 | 2005 | France | GenBank | C1 |
| AM236972 | 2005 | France | GenBank | C1 |
| AM236970 | 2005 | France | GenBank | C1 |
| AM236957 | 2005 | France | GenBank | C1 |
| AM236956 | 2005 | France | GenBank | C2 |
| AM236953 | 2005 | France | GenBank | C2 |
| AM236918 | 2005 | France | GenBank | C2 |
| AF317694 | 2000 | Sweden | GenBank | Metcalf (Outgroup) |
| AF081331 | 1955 | USA | GenBank | Metcalf (Outgroup) |
| PP891443 | 2022 | China | This study | C2 |
| PP891442 | 2022 | China | This study | C2 |
| PP891441 | 2022 | China | This study | C2 |
| PP891440 | 2019 | China | This study | C2 |
| PP891439 | 2019 | China | This study | C2 |
| PP891438 | 2019 | China | This study | C2 |
| PP891437 | 2019 | China | This study | C2 |

USA: United States of America; UK: United Kingdom.
